# Stress-related biomolecular condensates in plants

**DOI:** 10.1093/plcell/koad127

**Published:** 2023-05-10

**Authors:** Jorge Solis-Miranda, Monika Chodasiewicz, Aleksandra Skirycz, Alisdair R Fernie, Panagiotis N Moschou, Peter V Bozhkov, Emilio Gutierrez-Beltran

**Affiliations:** Institutode Bioquimica Vegetal y Fotosintesis, Consejo Superior de Investigaciones Cientificas (CSIC)-Universidad de Sevilla, 41092 Sevilla, Spain; Biological and Environmental Science and Engineering Division, Center for Desert Agriculture, King Abdullah University of Science and Technology, Thuwal 23955-6900, Saudi Arabia; Boyce Thompson Institute, Cornell University, Ithaca, NY 14853, USA; Max-Planck-Institute of Molecular Plant Physiology, 14476 Potsdam-Golm, Germany; Department of Plant Biology, Uppsala BioCenter, Swedish University of Agricultural Sciences and Linnean Center for Plant Biology, 75007 Uppsala, Sweden; Department of Biology, University of Crete, Heraklion 71409, Greece; Institute of Molecular Biology and Biotechnology, Foundation for Research and Technology-Hellas, Heraklion 70013, Greece; Department of Molecular Sciences, Uppsala BioCenter, Swedish University of Agricultural Sciences and Linnean Center for Plant Biology, 75007 Uppsala, Sweden; Institutode Bioquimica Vegetal y Fotosintesis, Consejo Superior de Investigaciones Cientificas (CSIC)-Universidad de Sevilla, 41092 Sevilla, Spain; Departamento de Bioquimica Vegetal y Biologia Molecular, Facultad de Biologia, Universidad de Sevilla, 41012 Sevilla, Spain

## Abstract

Biomolecular condensates are membraneless organelle-like structures that can concentrate molecules and often form through liquid-liquid phase separation. Biomolecular condensate assembly is tightly regulated by developmental and environmental cues. Although research on biomolecular condensates has intensified in the past 10 years, our current understanding of the molecular mechanisms and components underlying their formation remains in its infancy, especially in plants. However, recent studies have shown that the formation of biomolecular condensates may be central to plant acclimation to stress conditions. Here, we describe the mechanism, regulation, and properties of stress-related condensates in plants, focusing on stress granules and processing bodies, 2 of the most well-characterized biomolecular condensates. In this regard, we showcase the proteomes of stress granules and processing bodies in an attempt to suggest methods for elucidating the composition and function of biomolecular condensates. Finally, we discuss how biomolecular condensates modulate stress responses and how they might be used as targets for biotechnological efforts to improve stress tolerance.

## Teaser

This review describes the mechanism, regulation, composition, and properties of stress-related biomolecular condensates in plants.

## Introduction

Intracellular compartmentalization is integral to cellular function. In addition to conventional membrane-bound organelles, 2- or 3-dimensional compartments composed of multiple proteins, RNA molecules, and small-molecule ligands but lacking delineating lipid membranes offer an additional mechanism for intracellular organization (Gomes and Shorter 2019). Historically, these membraneless compartments have been termed ribonucleoprotein (RNP) granules, cellular bodies, membraneless bodies, or simply cellular aggregates. Recently, the unifying term biomolecular condensates has been coined to describe their capacity to spatially concentrate biomolecules (Banani et al. 2017).

The driving force for biomolecular condensate formation in many cases is biophysical in nature and is known as liquid-liquid phase separation (LLPS), whereby a solution separates into 2 (or more) phases (Emenecker et al. 2020; Pappu 2020). The first direct evidence demonstrating LLPS in cells was provided for P-granules in germ cells of the nematode *Caenorhabditis elegans*. P granules show liquid-like properties, such as fusion with one another and spontaneous exchange of their components with the cytoplasm (Brangwynne et al. 2009). After this seminal example, a considerable number of follow-up studies showed that many intracellular bodies exhibit similar behavior, including Lewy bodies, stress granules (SGs), processing bodies (PBs), frodosomes, purinosomes, bacterial RNP bodies, and FLOE1 granules (Cohan and Pappu 2020; Dorone et al. 2021; Hardenberg et al. 2021; Pedley et al. 2022). Notably, the formation of phase-separated condensates has been reported in the nucleus, cytoplasm, membranes, and chloroplasts (in plants) and has been implicated in a plethora of cellular programs that include gene expression, mRNA biogenesis, cell signaling, and metabolism (Fare et al. 2021; Londono Velez et al. 2022; Alberti and Hyman 2021). However, even though the number of studies on condensates has increased substantially in recent years, the mechanisms regulating their assembly remain largely unclear.

An emerging theme is that biomolecular condensates are major players during stress. In fact, the formation of stress-induced condensates has been described in response to a wide variety of stresses, indicating that their assembly is a common pathway invoked upon stress perception (Glauninger et al. 2022). The compartmentalization of proteins into stress-induced condensates is assumed to be an early event during stress response and exerts a cytoprotective role. In this context, the formation of SGs, one of the best-characterized stress-induced condensates in all eukaryotes, is involved in posttranscriptional regulation and translational control in response to stress (Youn et al. 2019). In addition to SGs, other condensates can also increase in number and/or size under stress, including PBs, plant small interfering RNA (siRNA) bodies, or yeast G-bodies (Martinez-Perez et al. 2017; Fuller et al. 2020). Despite growing interest in understanding the functional relevance of these assemblies, the composition of condensates and, more importantly, the mechanisms regulating their formation remain largely unknown. In plants, knowledge of stress-related condensates is still scarce. Nonetheless, recent studies have started to shed light on the molecular composition of a subset of stress-related condensates.

In this review, we focus on representative cytoplasmic stress-related condensates to provide a state-of-the-art overview of the mechanisms and regulation of phase-separated condensates and summarize the current knowledge of their composition and organization. Then we focus on a few selected examples of LLPS-formed condensates with important functions in stress signaling and acclimation. Special attention is paid to the unknowns in plant biology and why the field is lagging behind nonplant models. We further suggest research directions for elucidating the physiological roles of biomolecular condensates and review methodologies by which these could be realized.

## Principles governing the phase separation of condensates

**Figure 1.**
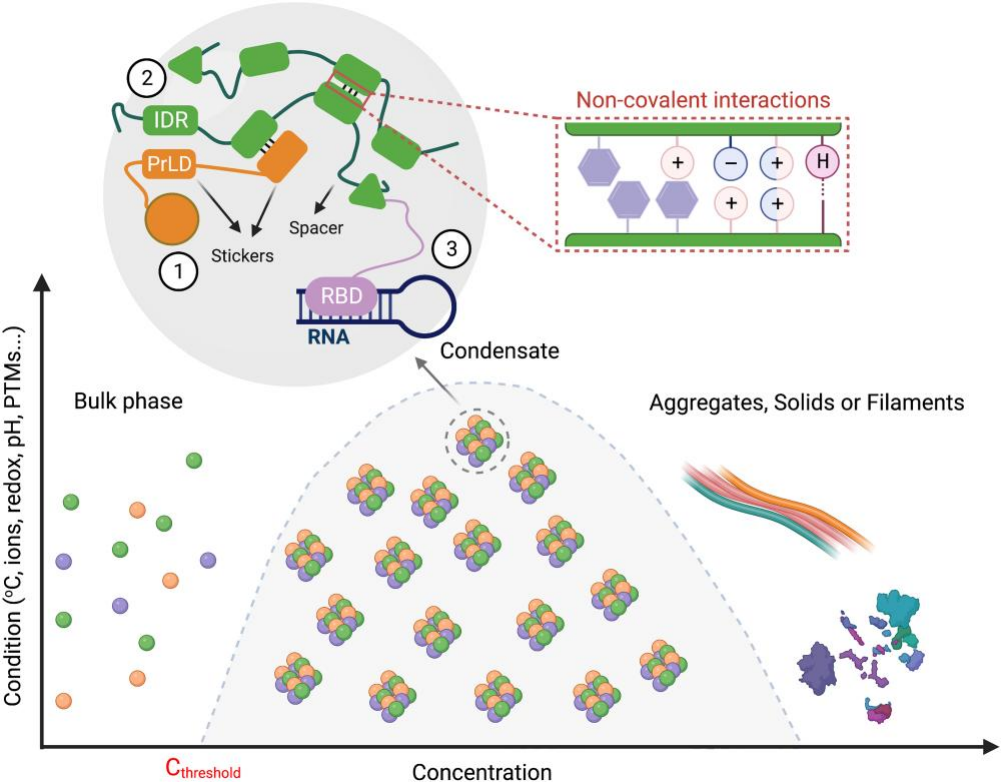
Diagram of the major principles underlying biomolecular condensate formation. A certain protein concentration (dependent on various factors, such as temperature, redox state, pH) enables homotypic or heterotypic interactions between sticker domains (e.g. protein 1–protein 2 interaction on the diagram). When reaching a system-specific threshold concentration (*C*_threshold_), the entire system undergoes phase separation into 2 phases. The “stickiness” (or multivalency) depends on the attraction between residues usually provided by so-called IDRs (e.g. PrLDs or LCDs). Phase separation driven by IDR–IDR interactions can be mediated by noncovalent interactions (boxed area) that include π (aromatic ring)–π, cation (+) –π, charge (−)–charge (+), dipole (±)–dipole (±), or hydrogen bonds (H). Folded domains or nucleic acids also mediate phase separation (e.g. protein 3 with an RNA-binding domain [RBD], in the diagram). Given enough time or at high concentrations, condensates may form filaments/aggregates with solid-like material properties. Created with BioRender.com.

Biomolecular condensates are assembled in many instances via LLPS, which leads to the formation of a dense phase with a high concentration of biomolecules surrounded by a dilute phase (Millar et al. 2023). Phase separation is promoted by an increase in the concentration of biomolecules and mediated by changes in the intracellular environment (e.g. temperature, redox state, pH, etc.), as summarized in Fig. 1. It has been proposed that LLPS might serve as a mechanism for the organization of biomolecules to regulate key biochemical functions (Fare et al. 2021; Musacchio 2022). Therefore, not surprisingly, the assembly of biomolecular condensates is tightly regulated, and its misregulation has been related to diseases such as cancer, neurodegeneration, or aging-associated disorders (Spannl et al. 2019; Conti and Oppikofer 2022; Chung et al. 2023). Understanding the general principles governing the phase separation of condensates and how they are organized and structured is critical to better understand their role in cell fate decisions and physiology.

### Multivalency-driven phase separation

Biomolecular LLPS relies on multivalency, meaning that the components of condensates can undergo multiple and simultaneous inter- or intramolecular interactions between homotypic or heterotypic molecules (Han et al. 2012; Li et al. 2012). The multivalency and affinity in biomolecular condensate formation can be relatively well explained by the stickers-and-spacers model in which biomolecular condensates form by reversible sticker-sticker interactions (Abyzov et al. 2022). While the stickers are responsible for the interactions driving condensation and thus biomolecular condensate formation, the intervening spacers connect the stickers and provide necessary flexibility (Fig. 1). Stickers can be made of folded domains, intrinsically disordered regions (IDRs), including low complexity domains (LCDs), as well as short linear amino-acid motifs (Mittag and Parker 2018). Many proteins with a high propensity to form condensates are enriched in IDRs and as such have gained significant attention as drivers of LLPS (Fig. 1) (Banani et al. 2017; Musacchio 2022). For example, a prion-like domain, a form of LCD, in FLOWERING TIME CONTROL A of Arabidopsis (*Arabidopsis thaliana*), can form nuclear condensates, showing the importance of intrinsic disorder in LLPS of plant condensates (Fang et al. 2019b).

Recent studies have pointed to a particularly important role for charge-charge, dipole-dipole, charge-π, π-π, and hydrogen bonds in enabling IDRs to phase separate (Fig. 1) (Li et al. 2018; Murthy et al. 2019; Krainer et al. 2021). Charge-π and π-π are types of noncovalent interaction involving aromatic rings (Meyer et al. 2003). Hence, tyrosine (aromatic) and arginine (charged) residues were shown to be necessary for the LLPS of a number of proteins, including Fused to sarcoma (FUS), the RNA helicase LAF-1, heterogeneous nuclear RNP A1, and Dead-box helicase 4 in mammalian cells (Nott et al. 2015; Vernon et al. 2018; Wang et al. 2018; Schuster et al. 2020). Although the mechanisms governing LLPS are still poorly studied in plants, it was reported that a tyrosine residue array situated in an LCD region of Arabidopsis RNA-BINDING GLYCINE-RICH PROTEIN D2 (RBGD2) and RBGD4 promotes their temperature-dependent LLPS during SG formation, demonstrating unsurprising conservation for the role of π systems in biomolecular condensation across kingdoms (Zhu et al. 2022).

### Regulation of condensate assembly

**Figure 2.**
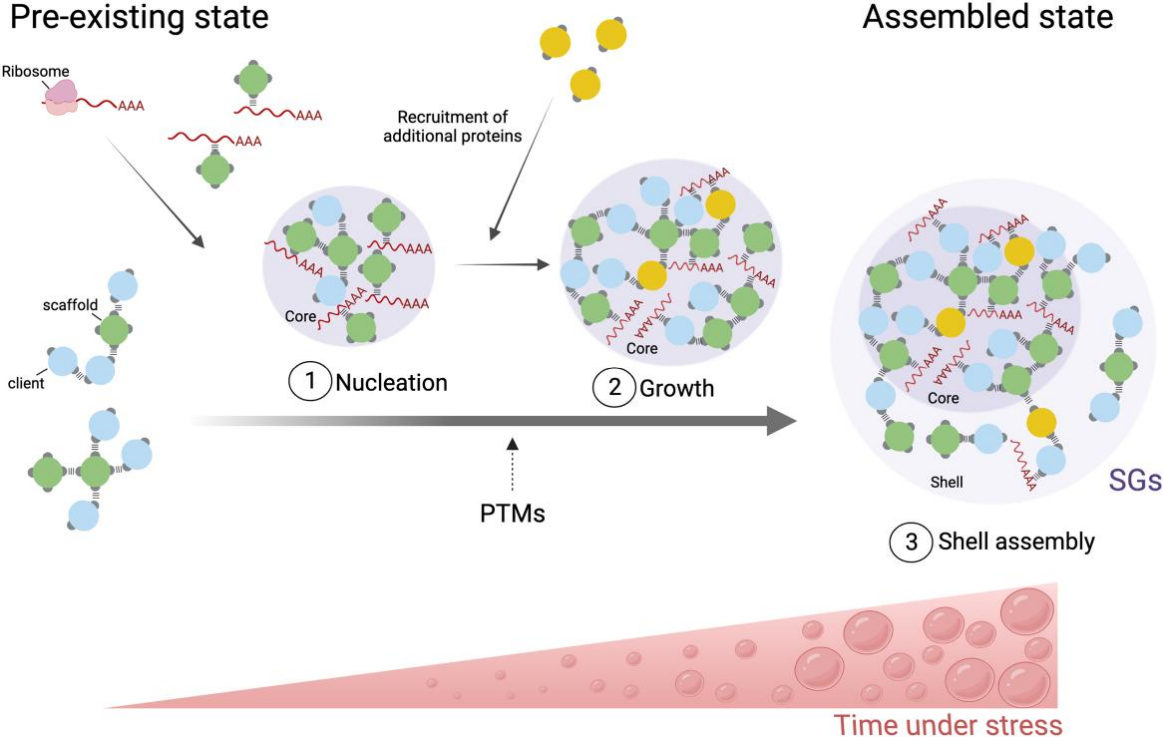
Basic principles of LLPS in the assembly of SGs. SGs are believed to assemble through 3 major steps: (1) nucleation, (2) growth, and (3) shell assembly. Stress inhibits translation, which triggers release of mRNAs from the polysomes, which, together with RBPs, promotes nucleation via LLPS. Next, specific recruitment of additional SG components facilitates core growth (2) and thereafter shell assembly (3). The phase-separating biomolecules (usually proteins) can be categorized as scaffold or clients. In this figure, scaffold and client components are represented as spheres (green for scaffolds and blue for clients) with attractive sites on their surface (gray patches). Each patch (valency) allows a protein to participate in one attractive protein–protein or RNA–protein interaction. In the absence of stress, SG components may exist as preformed protein complexes (pre-existing or standby state) serving as seeds for rapid assembly (Gutierrez-Beltran et al. 2021). Upon stress, these complexes may facilitate the recruitment of RNAs and other proteins into phase-separated condensates that become microscopically discernible fluorescent foci if properly labeled. This phase separation may be modulated by posttranslational modifications. Created with BioRender.com.

Although studied mostly in yeast (*Saccharomyces cerevisiae*) and mammalian cells, the best-understood model of stress-induced biomolecular condensation is that of SGs because these condensates form in response to exogenous stimuli and are not constitutively present in the cell. SGs are RNA-protein condensates with biphasic organization, comprising stable cores surrounded by a more dynamic shell (Wheeler et al. 2016). The assembly of SGs is likely a multistep, highly controlled program that can be briefly described by 3 consecutive steps: first, the formation of a dense stable SG core via LLPS (nucleation); second, the growth of the core by the recruitment of additional SG components—so-called clients (growth); and third, accumulation of proteins into a peripheral shell (shell assembly) (Fig. 2) (Banani et al. 2017; Markmiller et al. 2018; Kosmacz et al. 2019; Cirillo et al. 2020). An important question is to what extent other types of biomolecular condensates, especially in plants, form through the same sequence of events as those described for yeast and mammalian SGs. Several recent findings in mammalian systems support the idea that PBs, a type of cytosolic biomolecular condensates functionally linked to SGs and mainly involved in mRNA degradation, may implicate a similar principle of multi-step assembly. Indeed, several findings have shown that PBs contain densely populated subdomains, including a relatively stable core, pointing to the existence of a differential organization within PBs (Souquere et al. 2009; Hubstenberger et al. 2017). In addition, typical PB core proteins can phase separate in vitro, suggesting that the nucleation step might be involved in the PB biogenesis in vivo (Schutz et al. 2017; Luo et al. 2018). However, unlike SGs, PBs exist at a basal level under unstressed (normal) conditions and are strongly induced in response to stress, indicating that the pathways of PB and SG assembly as a whole must be different.

Although many mechanistic details of SG assembly remain unclear, all of the proposed models converge on the view that nucleation is the key step (Glauninger et al. 2022). Growing evidence suggests that stress-induced RNA-RNA, RNA-IDR, and IDR-IDR interactions initiate the nucleation step (Protter and Parker 2016a; Ditlev et al. 2018; Mittag and Parker 2018; Sanders et al. 2020). Posttranslational modifications of SG-associated proteins, such as methylation, ubiquitination, or phosphorylation, also contribute to SG nucleation (Fig. 2) (Protter and Parker 2016b). Despite a recent study in plants showing that the phosphorylation of the Arabidopsis SG component GLYCINE-RICH RNA-BINDING PROTEIN 7 (GRP7) is required for its nucleation (Xu et al. 2022), the role of GRP7 in SG formation remains unclear. Although posttranslational modifications affect SG nucleation, how the 2 events are co-regulated upon stress perception remains an open question.

### Molecular organization of biomolecular condensates

Upon formation, biomolecular condensates can increase in complexity through an increase in protein, RNA, or other molecules (including metabolites) contents if the shell (or a similar less dense phase) is permeable to these molecules (Mitrea et al. 2022). The molecular composition of condensates is tightly controlled; some components are constitutive, whereas others are only transiently recruited under certain conditions. The scaffold-client model can explain this differential recruitment. Scaffolds are multivalent molecules (usually proteins) stably associated with biomolecular condensates and essential for assembly, whereas clients are transiently associated with condensates and likely recruited by scaffolds (Fig. 2) (Ditlev et al. 2018). In contrast to clients, scaffolds are considered to be drivers of phase separation. However, classification into these 2 classes has some limitations because scaffold and client proteins may switch roles (Ditlev et al. 2018; Riback et al. 2020). Furthermore, biomolecular condensate formation may be modular, wherein a client may be converted to a scaffold to bring in other clients. These secondary scaffolds may be important for adding accessory proteins, thereby modulating the functionalities of the biomolecular condensates.

The molecular mechanisms by which scaffolds recruit clients and how they promote phase separation are still a matter of speculation. Growing evidence suggests that multiple folded domains (e.g. the SRC homology 3 domains in the noncatalytic region of tyrosine kinase) or IDRs from scaffolds contribute to generating a network of interactions between proteins or proteins and RNA, thus facilitating recruitment and LLPS (Banani et al. 2017). The best such example is probably the scaffold protein Ras GTPase-activating protein-binding protein 1 (G3BP1), whose IDRs interact with RNAs to facilitate the assembly of mammalian SGs (Yang et al. 2020). In contrast to mammals, only a few proteins required for the assembly of biomolecular condensates have been described in plants. A recent study revealed that a disordered region of a multivalent protein tudor staphylococcal nuclease (TSN) provides a docking platform for interaction with a large pool of other intrinsically disordered proteins. In addition, this region was required for the recruitment of some of these intrinsically disordered proteins to cytoplasmic foci upon stress (Gutierrez-Beltran et al. 2021).

To date, the layered organization of specific condensates such as SGs is a rather well established albeit oversimplified notion (Protter and Parker 2016a; Fare et al. 2021). Advanced microscopy techniques have become key tools for studying the molecular organization of biomolecular condensates. For example, super-resolution microscopy revealed that mammalian G3BP1 forms a dense core surrounded by a more dilute shell (Jain et al. 2016). An intriguing type of organization was observed for biomolecular condensates formed by the AUXIN RESPONSE FACTOR (ARF) family of transcription factors in the cytoplasm of Arabidopsis root cells (Rogg and Bartel 2001). Using fluorescence correlation spectroscopy, ARF condensates were demonstrated to show an inverse organization, compared with SGs, with the more stable layer being at the condensate exterior, that is, constituting the shell (Powers et al. 2019). ARF sequestration into cytoplasmic condensates blocks its entry into the nucleus, thus decreasing auxin responsiveness (Powers et al. 2019). Whether the stable shell of ARF condensates mediates the blockage of nuclear entry remains to be seen. This organization found in ARF condensates, however, is not unique to plants and has been observed for condensate-like structures formed in prokaryotes and known as bacterial microcompartments (Kerfeld et al. 2018). Despite new technical advances, a molecular topology of multiple components inside the condensates remains elusive.

### Liquid-solid properties of biomolecular condensates during stress

Non-plant biomolecular condensates can harden (i.e. become less liquid and resemble a more solid state) or increase in size over time, especially in vitro when the components reach equilibrium. Among other mechanisms, Ostwald ripening contributes to these changes by driving the disappearance of small condensates via their dissolution and deposition of their now released components into pre-existing larger biomolecular condensates (Dine et al. 2018). The driving force for Ostwald ripening is the difference in solubility between small and large biomolecular condensates. It is thus expected that given enough time, a single biomolecular condensate would remain, akin to the separation of oil and vinegar in salad dressing, where we see gradual coarsening of oil droplets. This state, apart from Ostwald ripening, can be also driven by collisions and fusions between distinct condensates.

These events might be physiologically relevant for plant condensates as well because Ostwald ripening may drive the formation of the eukaryotic pyrenoid in the unicellular alga *Chlamydomonas reinhardtii*, which ends up forming a single droplet (Freeman Rosenzweig et al. 2017). Yet, for reasons not completely understood, most condensates do not become a singular entity in cells. During stress, however, biomolecular condensates do become larger, suggesting that the mechanisms restricting condensate sizes might be suppressed. For example, PBs and SGs increase in size during stress progression in Arabidopsis (Liu et al. 2023). A lack of some scaffold proteins may also affect the size of the condensates, a phenomenon documented in TSN-deficient Arabidopsis cells (Gutierrez-Beltran et al. 2015).

The presence of a tight core and a loose shell in some types of biomolecular condensates suggests that they can be viewed as ensembles of materials with varying properties. Indeed, a key feature of biomolecular condensates is that they can be both viscous (a hallmark of liquids) and elastic (as observed in solids), a phenomenon known as viscoelasticity (Bergeron-Sandoval and Michnick 2018). Once the material is deformed, it may never return to its original shape. Yet, this model for condensate organization comes from the animal research field, where the material properties of biomolecular condensates have been relatively well studied, including under stress conditions. Biomolecular condensates were shown to behave more like an elastic solid or a viscous liquid, depending on various parameters, including shear stress applied to a condensate as well as its age and size (Shen et al. 2020).

During the aging of biomolecular condensates (also known as maturation or growth), the accumulation of various protein conformations causes an imbalance in intermolecular interactions (Garaizar et al. 2022). These metastable conformations become more important with time, leading to the assembly of liquid-core/gel-shell (e.g. ARFs) or gel-core/liquid-shell (e.g. SGs) architectures. Importantly, changes in the architecture of biomolecular condensates can be attributed to perturbations in their turnover, allowing them to stay around longer and age (Yamasaki et al. 2020), similar to phenomena linked to chronological aging and various human neurodegenerative diseases (Patel et al. 2015; Alberti and Hyman 2016). Interestingly, how these transitions in material properties are modulated by stresses remains elusive but would be important to understand because these transitions may affect the residence time of key regulatory proteins in biomolecular condensates.

### Biomolecular condensates and membranes

Back in 2012, the pioneering study of Li and coworkers showed that biomolecular condensates can interface with membranes in animal cells (Li et al. 2012). Biomolecular condensates can form films on membranes that extend laterally and are characterized by smooth and circular boundaries (Yuan et al. 2021). Optically, such thin films resemble membrane patches (Vequi-Suplicy et al. 2010; Kusumaatmaja et al. 2021). These features suggest that many membrane-bound patches may be as yet unidentified biomolecular condensates. Given that the plasma membrane is the first cellular barrier to encounter the environment and thus encounter stress, research in this direction is especially important.

In plants, an example of a condensate that wets membranes is that of the lipid transferase Sec14-HOMOLOG 8 (SFH8) (Liu et al. 2022). Membranes facilitate the condensation of SFH8 by lowering the threshold concentration by 50-fold, likely through interactions with phosphatidylinositol lipids (Liu et al. 2022), as has been reported for other proteins in nonplant species (Case 2022). Many phosphatidylinositol lipids are subjected to regulation by stress, raising the question of whether biomolecular condensates might be regulated by alterations in membrane lipids. In animals, phosphatidylinositol lipids mediate the phase separation of argonaut proteins (AGOs) on the endoplasmic reticulum (Gao et al. 2022). These lipids are highly responsive to stress conditions (Hou et al. 2016); thus it would be interesting to assess their potential role in biomolecular condensation in plants.

Recently, membrane wetting by DECAPPING PROTEIN 1 (DCP1), a major component of PBs, was shown to lead to PB dissolution (Liu et al. 2023). DCP1 recruitment at the plasma membrane partially depends on an actin nucleating complex known as SCAR-WAVE. In turn, DCP1–SCAR/WAVE forms a condensate that promotes actin nucleation. PB dissolution decreased during heat stress, which in principle could affect the global transcriptome profile of the cell and thus stress tolerance. The link between condensation at the plasma membrane and the transcriptome merits further investigation, especially during stress. Furthermore, the above principles of condensation may allow for tight control of receptor clustering, with as yet not understood implications for stress responses, especially immune responses as in animal cells (Su et al. 2016).

## Establishing the properties of stress-related biomolecular condensates in plants

### Proteome

**Figure 3.**
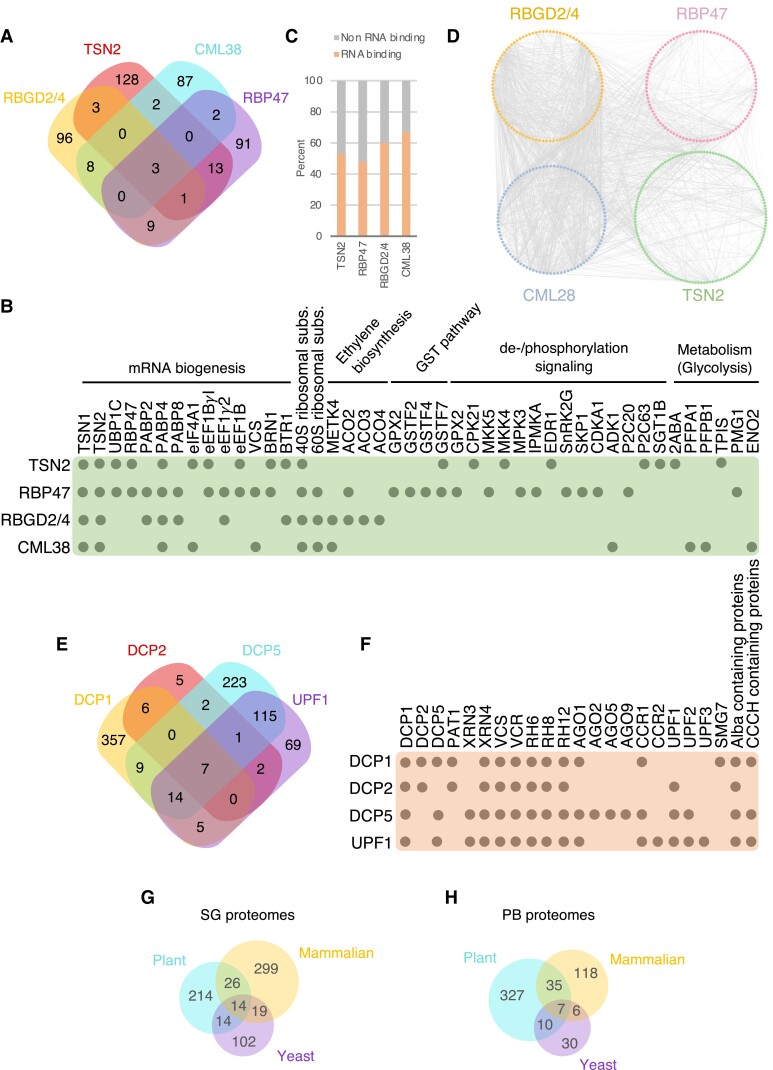
Proteomic analysis of plant SGs and PBs. **A**) Venn diagram showing the extent of overlap among interactomes for 4 different SG-associated proteins (RGBD2/4, TSN2, RBP47, and CML38) under stress. **B**) A subset of common and specific interactors of the proteins in (**A**). **C**) Percentage of RNA-binding proteins found in the 4 interactomes. **D**) Protein–protein interaction networks among the RGBD2/4, TSN2, RBP47, and CML38 interactomes. **E**) Venn diagram showing the extent of overlap among interactomes for 4 different PB-associated proteins (DCP1, DCP2, DCP5, and UPF1) under stress. **F**) A subset of common and specific interactors of the proteins in (**E**). For complete lists of Arabidopsis SG and PB proteome components, see Supplemental Data Sets S1 and S2. **G**) Venn diagram showing the extent of overlap among plant, mammalian and yeast SG proteomes. **H**) Venn diagram showing the extent of overlap among plant, mammalian and yeast PB proteomes.

In nonplant models, characterizing condensate proteomes has significantly aided in understanding condensate dynamics, regulation, and functions. Similar attempts in plants are still in their infancy, and most of the available information concerns mass-spectrometry analysis of interactomes for SG-resident proteins in Arabidopsis. The degree of similarity among such interactomes can be used as a proxy for evaluating inherent variability among SG proteomes. Here we provide a comparative analysis of 3 heat stress–induced interactomes [namely of TSN2 (Gutierrez-Beltran et al. 2021), RBGD2 and RBGD4 (Zhu et al. 2022), and RNA-BINDING PROTEIN 47 (RBP47) (Kosmacz et al. 2019)] and the hypoxia-induced interactome of CALMODULIN-LIKE PROTEIN 38 (CML38) (Lokdarshi et al. 2015) (Fig. 3A; Supplemental Data Sets S1 and S2). Only 3 proteins were shared by all 4 interactomes: TSN1, TSN2 (in one case as a bait), and POLY-A BINDING PROTEIN 4. From this group, Arabidopsis TSN proteins appear to be a central hub, consistent with their scaffolding role in SGs (Gutierrez-Beltran et al. 2015; 2021; Maruri-Lopez et al. 2021) (Fig. 3B). In addition to these proteins, we observed an overlapping group comprising well-defined SG components, including RBP47, OLIGOURIDYLATE-BINDING PROTEIN 1 C, other PABPs, different ribosomal subunits (40 and 60S), and several translation initiation and elongation factors (eIFs, eEFs) (Fig. 3B). Notably, similar to mammals and yeast, plant SG interactomes display a dense network of protein–protein interactions and are enriched for RNA-binding proteins (RBPs) (Jain et al. 2016; Marmor-Kollet et al. 2020) (Fig. 3, C and D).

Comparison among the 4 plant interactomes also demonstrates that conserved core SG proteins coexist with other cell- and stress type-specific components, suggesting that SGs are multifunctional condensates with highly heterogenous protein contents (Fig. 3B). For example, enzymes involved in protein dephosphorylation or phosphorylation, ethylene biosynthesis, the glutathione-S-transferase pathway, or glycolysis are overrepresented in some of the SG protein interactomes but missing in others. There is now growing evidence that the incorporation of enzymes in biomolecular condensates can increase their catalytic activity through concentration, conformational changes, or other mechanisms (Peeples and Rosen 2021; Mountourakis et al. 2023).

SGs are functionally linked with PBs, and both have been suggested to exchange proteins and RNAs (Jang et al. 2020; Maruri-Lopez et al. 2021). Although PBs are constitutively present in the cell, they can increase in number and size during stress (Gutierrez-Beltran et al. 2015; Chicois et al. 2018; Jang et al. 2020). In contrast to mammals, the composition of plant PBs is not well defined. We compared published interactomes of the well-known PB components DCP1, DCP2, DCP5, and UP-FRAMESHIFT 1 (UPF1) (Chicois et al. 2018; Schiaffini et al. 2022; Liu et al. 2023) (Fig. 3E; Supplemental Data Set 2). We determined that similar to mammals, plant PBs accumulate mRNA decapping factors (DCP1, DCP2, DCP5, PROTEIN-ASSOCIATED WITH TOPOISOMERASE 1, and VARICOSE), 5′-3′ processing exonucleases (XRN3 and XRN4), nonsense-mediated mRNA decay factors (UPF1, UPF2, UPF3, and SMG7), components of the microRNA pathway (AGO1, AGO2, AGO5, and AGO9), and RNA helicases (RH6, RH12, and RH12) (Fig. 3F). The accumulation of mRNA decay factors in PBs is in line with their canonical role in executing mRNA degradation. However, recent research suggests that thousands of mRNAs accumulate in human PBs to evade RNA decay (Hubstenberger et al. 2017). In agreement, ACETYLATION LOWERS BINDING AFFINITY 4 (ALBA4), ALBA5, and ALBA6 confer plant thermotolerance by stabilizing the mRNA of HEAT STRESS TRANSCRIPTION FACTORs (*HSF*s) in cytoplasmic biomolecular condensates, including PBs (Tong et al. 2022). Intriguingly, ALBA domain-containing proteins are enriched in all 4 plant PB interactomes available today (Fig. 3F).

To investigate how similar plant, mammalian, and yeast proteomes are, we used the eggNOG orthology database (Huerta-Cepas et al. 2019). The comparison of SG proteomes reveled that approximately 15% of proteins from plants are shared by mammalian or yeast SGs (Jain et al. 2016), including well-characterized SGs core proteins such as translation associated factors (e.g. PABP2/4/8 or eIF4A), RNA-binding proteins (e.g. the RNA-binding KH domain-containing protein HUA ENHANCER 4) or ribosomal subunits (e.g. RPS2) (Fig. 3G). In the case of PBs, the overlap group, which represents approximately 14% of all proteins, includes proteins involved in mRNA decay (e.g. DCP1, DCP2, DCP5, and UPF1) or RNA helicases (e.g. RH6, RH8, RH12) (Fig. 3H). These results show a compositional conservation in core components between kingdoms, which is consistent with the canonical role of both condensates in RNA metabolism (Youn et al. 2019; Kearly et al. 2022). However, many proteins from both SG and PB proteomes are kingdom specific, suggesting that each condensate might play additional roles that are fully dependent on the organism.

Compared with SGs and PBs, other plant cytoplasmic stress-related condensates are even more enigmatic in terms of their protein composition and architecture. For example, plant siRNA bodies play a role in siRNA amplification during stress and typically contain SUPPRESSOR OF GENE SILENCING 3 (SGS3) and RNA-DEPENDENT RNA POLYMERASE 6 (RDR6), explaining why these bodies are also named SGS3/RDR6 bodies (Martínez de Alba et al. 2015; Field et al. 2021). SGS3 is an RNA-binding protein that, together with RDR6, is necessary for the synthesis of double-stranded RNA templates for their subsequent processing into secondary siRNAs during stress. Although the dynamics and molecular composition of siRNA bodies remain largely unknown, phase separation of both SGS3 and RDR6 proteins is important for their assembly (Kim et al. 2021). Other proteins identified in siRNA bodies include Arabidopsis AGO7, the m^6^A demethylase ALKBH9B, and *Nicotiana benthamiana* calmodulin-like (Jouannet et al. 2012; Martinez-Perez et al. 2017).

### Transcriptome

In addition to proteins, RNAs are found in several types of biomolecular condensates, including SGs, PBs, and siRNA bodies. Therefore, these condensates are often referred to as RNP granules. Although SGs and PBs both contain nontranslating mRNAs, their fates in the 2 compartments were initially thought to be storage and degradation, respectively (Protter and Parker 2016a). However, recent studies now challenge this notion. RNA immunoprecipitation followed by sequencing analysis of mammalian SG RNAs revealed a subset of translationally active mRNAs (Mateju et al. 2020). Furthermore, a fluorescence-activated particle sorting demonstrated that mRNAs in PBs are translationally repressed but not degraded (Hubstenberger et al. 2017). Until recently, it was widely accepted that SGs and PBs are physically connected, continuously exchanging their mRNAs and proteins during stress. However, a single-mRNA imaging approach showed that, in contrast to proteins, very few mRNA molecules in fact shuttle between SGs and PBs during stress (Mateju et al. 2020). In further contrast to the mammalian and yeast systems, the RNA composition and the fate and role of individual mRNAs present in plant stress-induced condensates are emerging topics. It has been suggested that the localization of heat-induced transcripts in Arabidopsis SGs might promote the heat-stress response (Zhu et al. 2022). In agreement, the stabilization of *HSF* mRNAs in SGs and PBs was reported to facilitate thermotolerance (Tong et al. 2022). More research is, however, required to unravel the RNAs within and the mechanistic role of biomolecular condensates in translation and other RNA-dependent pathways during plant stress responses.

### Crosstalk among stress-induced cytoplasmic biomolecular condensates

**Figure 4.**
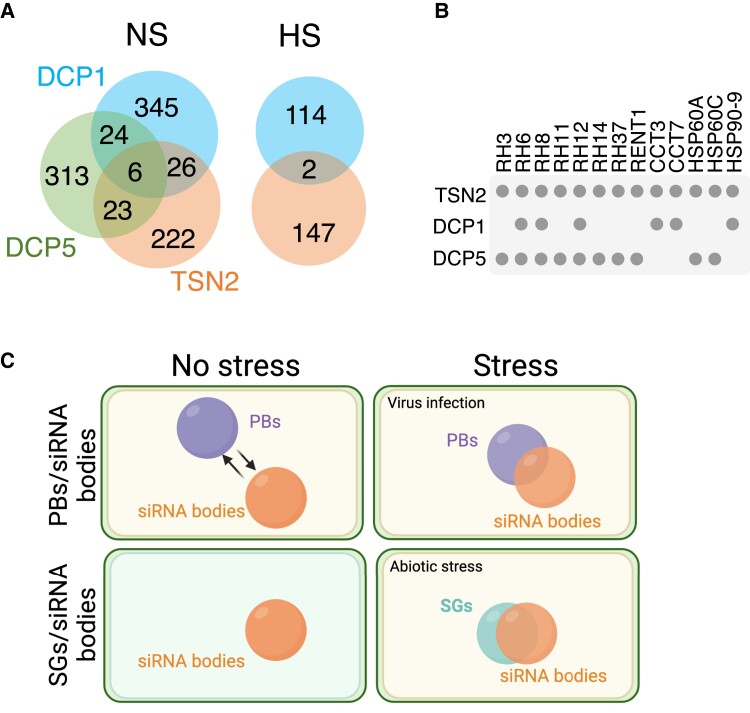
Crosstalk among plant SGs, PBs and siRNA bodies. **A**) Venn diagrams showing the extent of overlap among DCP1, DCP5 (both for PBs), and TSN2 (for SGs) interactomes under no-stress (NS) conditions (left) and between DCP1 and TSN2 interactomes under heat stress (HS). **B**) A subset of common and specific interactors of DCP1, DCP5, and TSN2 at the absence of stress. **C**) Diagram of the relationships among SGs, PBs, and siRNA bodies under no stress conditions and upon onset of stress. For complete lists of Arabidopsis SG and PB proteome components, see Supplemental Data Sets S1 and S2. Created with BioRender.com.

The bulk of SGs or other biomolecular condensates may exist as stable submicroscopic structures in the absence of stress in a pre-existing, standby state (Glauninger et al. 2022). Considering this notion and the fact that PBs are constitutively present in the cells, we compared the available interactomes of Arabidopsis SG and PB resident proteins (TSN2 vs DCP1 or DCP5, respectively) in the absence of stress and under heat stress to ask whether nucleation or growth of SGs and PBs engage similar proteins (Fig. 4A) (Chicois et al. 2018; Gutierrez-Beltran et al. 2021; Liu et al. 2023). In the absence of stress, the proteins shared by the PB- and SG-related interactomes included conserved condensate remodelers, such as protein chaperones (e.g. HEAT SHOCK PROTEIN 60 [HSP60] and HSP90 and T-COMPLEX PROTEIN [CCT]) and RNA and DNA helicases (e.g. DEA-box proteins or REGULATOR OF NONSENSE TRANSCRIPTS 1) (Fig. 4B). This finding suggests that SGs and PBs may use a similar set of scaffolding protein structures that do not grow further in the absence of stress.

In fact, the condensate remodelers DEAD-Box Helicase 6 and CCT, homologous to Arabidopsis RH6/RH8/RH12 and CCT proteins, respectively, have been described as key players in PB and SG assembly in mammalian and yeast cells (Ayache et al. 2015; Jain et al. 2016; Di Stefano et al. 2019). In mammals, pre-existing interactions among a subset of SG- and PB-associated proteins may act hierarchically as seeding scaffolds to recruit clients (proteins and mRNAs), thereby facilitating condensate growth (Youn et al. 2019). Whether plant SGs and PBs exploit a similar type of hierarchical relations in their preassembled state to potentiate subsequent growth and acquisition of the core shell organization is unknown and awaits studies. Once Arabidopsis cells perceive a heat stimulus, their SG and PB proteomes become more dissimilar, with only a very few proteins being in common (Fig. 4A). These observations suggest that favorable growth conditions suppress the identity of the SG and PB precursor protein complexes, whereas onset of stress facilitates their compositional and structural dichotomy.

Although SGs, PBs, and siRNA bodies have recently been recognized as major players in regulating the fate and function of cytoplasmic RNAs during plant stress responses, the interplay among these condensates remains obscure (Makinen et al. 2017). As discussed above, physical interaction and material exchange among these cytoplasmic condensates depends on the environmental conditions, which may also be involved in the establishment of their identity. In this regard, siRNA bodies colocalize with SG protein markers under hypoxia and heat stress (Fig. 4C) (Jouannet et al. 2012; Field et al. 2021), pointing to the possibility that mRNAs stalled in translation may accumulate in cytoplasmic condensates representing hybrids between SGs and siRNA bodies under abiotic stress in plants.

Whereas siRNA bodies appear to be compositionally distinct from PBs in the absence of stress, the 2 types of biomolecular condensates display a functional interrelationship (Fig. 4C) (Martínez de Alba et al. 2015). Indeed, it has been proposed that mRNA decapping of nonfunctional RNAs in Arabidopsis PBs prevents their entry into siRNA bodies, in which they would potentially be converted into siRNAs. Recent studies have shown a tight connection between siRNA bodies and PBs during viral infection (Fig. 4C). First, the RNA N6-methyladenosine demethylase AlKBH10B, which is required for viral RNA biogenesis, colocalized with the PB-associated proteins UPF1 and DCP1 (Li et al. 2017). More recently, it was shown that the association of PB components with the cauliflower mosaic virus might protect viral RNAs from siRNA body-dependent translational repression (Hoffmann et al. 2022). Although there is increasing evidence for molecular crosstalk among SGs, PBs, and siRNA bodies, a more exhaustive analysis is required to better understand shared and unique functions of these biomolecular condensates in plant stress biology.

## Biomolecular condensates as mediators and regulators of stress responses

### Condensates and gene expression

**Figure 5.**
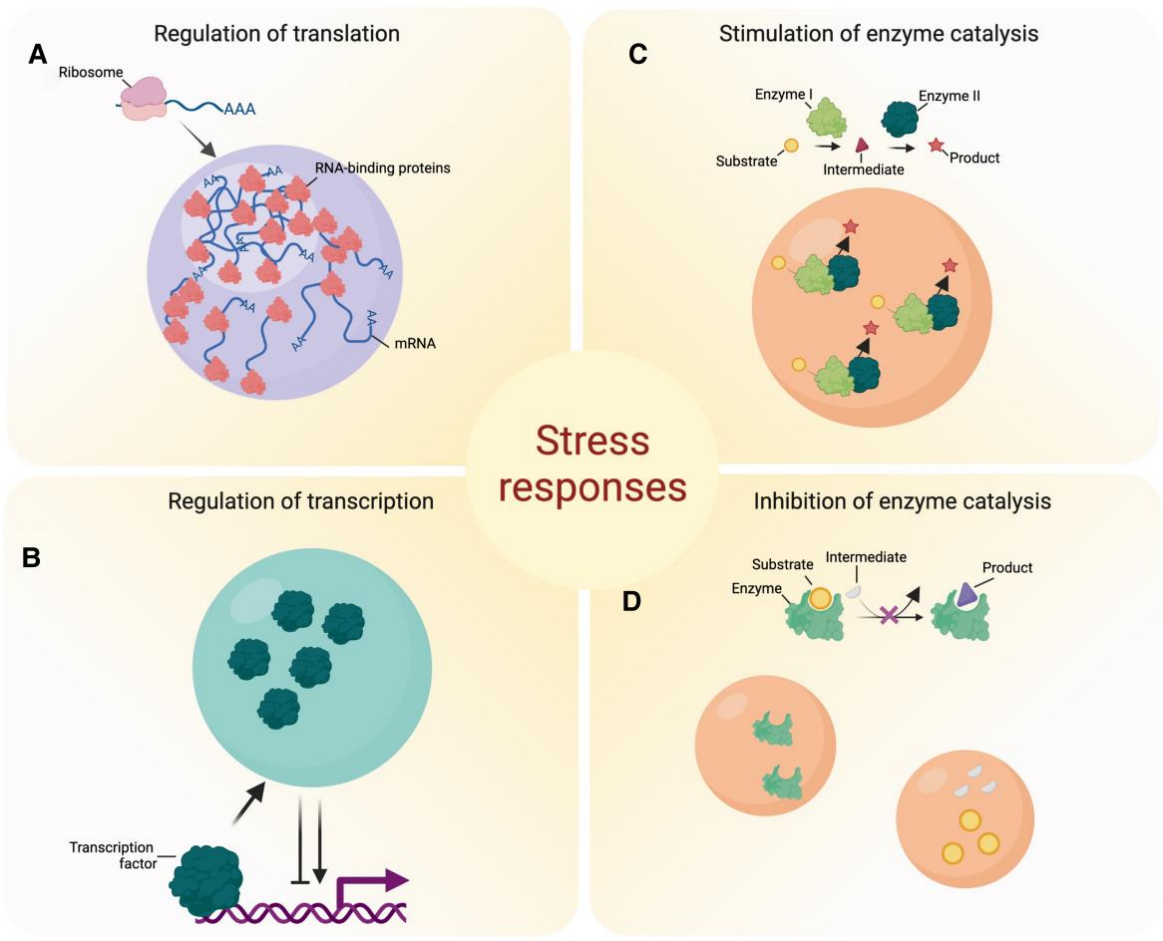
Biomolecular condensates play a key role in stress responses. **A**) The sequestration of transcription factors and regulators in condensates can regulate transcription, either promoting or inhibiting it. **B**) Condensates can either increase or decrease translation efficiency. **C**) Due to mass action, concentration of enzymes and substrates in the condensates can enhance catalysis or even allow formation of metabolons with improved efficiency. **D**) Condensates can inhibit enzymatic reactions and pathways in the dilute phase (e.g. cytosol) by sequestering enzymes, their ligands or substrates as well as metabolic intermediates. Inhibition of the reaction can also be achieved by separating different components of the common pathway (e.g. enzyme and substrate) via sequestration into different types of biomolecular condensates. Created with BioRender.com.

Considering that many proteins in biomolecular condensates bind RNA molecules, sequestration of these proteins within condensates may alter the translational landscape or other functions related to noncoding RNAs to favor cell survival and acclimation (Fig. 5A). For example, a DEAD-box RNA helicase (Ded1p)-dependent translational switch mechanism in yeast was suggested as a mediator of acclimation to heat stress (Iserman et al. 2020). In response to heat, Ded1p is targeted to SGs, where it is thought to initiate the scanning of mRNAs for housekeeping genes containing a structurally complex 5′ untranslated region to silence them and in this way promote the translation of stress-response RNAs with simpler 5′ untranslated regions (Iserman et al. 2020). Interestingly, RH20 is the Arabidopsis ortholog of Ded1p, but it is unclear whether it can modulate the translation of mRNAs from housekeeping genes under stress even though other RH proteins have been implicated in plant stress responses, presumably via their SG and/or PB localization (Chantarachot et al. 2020).

Apart from the direct role of biomolecular condensates in controlling the translational landscape through RNA sequestration, they can additionally be involved in the regulation of transcription (Fig. 5B). Plant GUANYLATE-BINDING PROTEIN (GBP)-LIKE GTPases (GBPLs) form biomolecular condensates in the nucleus to protect against infection and autoimmunity (Huang et al. 2021). GBPL3 defense-activated condensates assemble when GBPL1, a pseudo-GTPase, sequesters catalytically active GBPL3 under normal conditions but is displaced by GBPL3 LLPS when it enters the nucleus following immune cues. This altered GBPL3 defense-activated condensate formation impairs the recruitment of GBPL3 and salicylic acid (SA)-associated Mediator subunits to the promoters of *CALMODULIN-BINDING PROTEIN 60 g* and *SYSTEMIC ACQUIRED RESISTANCE DEFICIENT 1*, which encode master transcription factors involved in immunity (Wang et al. 2011).

Interestingly, some biomolecular condensates may switch function upon translocation from the nucleus to the cytoplasm. Pathogen effector-triggered immunity often leads to programmed cell death, which is restricted by NONEXPRESSER OF PR GENES 1 (NPR1), an activator of SA-mediated systemic acquired resistance. NPR1 promotes cell survival by targeting substrates for ubiquitination and degradation through the formation in the cytoplasm of SA-induced NPR1-rich condensates (SINCs) (Zavaliev et al. 2020). The SINCs are enriched in stress response proteins, including nucleotide-binding leucine-rich repeat immune receptors, oxidative and DNA damage response proteins, and protein quality control machinery. The condensation of NPR1 into cytoplasmic SINCs promotes the formation of a complex between NPR1 and the E3 ligase CULLIN 3 to ubiquitinate SINC-localized substrates, such as ENHANCED DISEASE SUSCEPTIBILITY 1 and the stress-related transcription factors from the WRKY family that positively affect effector-triggered immunity, thereby promoting survival. Importantly, this cytoplasmic function of NPR1 complements its transcriptional role in the nucleus. How and whether SINCs interplay with other cytoplasmic condensates, including SGs, PBs, and siRNA bodies, remains unknown.

### Condensates and metabolism

The presence of biomolecular condensates has frequently been postulated to explain cellular features of metabolism in animal and microbial systems (Robinson et al. 1987; Sweetlove and Fernie 2013; 2018). In fact, stress-induced sequestration of enzymes into condensates was proposed to mediate or regulate biochemical reactions under adverse conditions in mammals (Fig. 5, C and D) (Peeples and Rosen 2021). Hence, biomolecular condensates may recruit enzymes and their substrates, thereby acting as a core promoting a specific biochemical reaction (Fig. 5C). Indeed, the mechanistic dissection of increased enzymatic rate in a phase-separated compartment was recently demonstrated for the SUMOylation enzyme cascade in the mammalian system. SUMOylation rates increased by up to 36-fold in molecular condensates (Peeples and Rosen 2021), resembling the increases in reaction rates reported for other enzyme-enzyme assemblies (Zhang et al. 2020). Moreover, Peeples and Rosen found that the increased SUMOylation efficiency was due to increased concentrations enhancing the mass action as well as through multivalent hetero- or homotypic interactions that may evoke conformational changes affecting substrate *Km*. In plants, comparable direct evidence has been provided by the analyses of SG proteomes (described above), whereas indirect evidence comes from the evaluation of spatial allocation of metabolites in the cell by means of nonaqueous fractionation and by analysis of the metabolic fate of heavy label isotopes (Szecowka et al. 2013).

Biomolecular condensates in cells are often rich in catalytically active enzymes from metabolic pathways (Fig. 3B) (Ouazan-Reboul et al. 2021). Their formation and disassembly are dynamically responsive to environmental conditions and stimuli. In a subset of these assemblies, metabolites may be channeled between sequential enzymes: that is, the product of one enzyme is transferred to the next enzyme in the pathway without equilibrating with the bulk aqueous phase of the cell (Fig. 5C). In such cases, the assemblies are known as metabolons. Since this finding, assemblies of consecutive enzymes have been observed in a wide variety of metabolic pathways (Shen 2015; Sweetlove and Fernie 2018). For example, the aggregation of enzymes of the phenylpropanoid pathway in plants highlights substrate channeling between phenyl ammonia-lyase and cinnamate 4-hydroxylase (Rasmussen and Dixon 1999; Achnine et al. 2005). Many results from studies of this pathway [see for example those in (Burbulis and Winkel-Shirley 1999; Crosby et al. 2011)] are in keeping with a recent suggestion that the presence of enzyme-enzyme assemblies is likely important in directing flux via various branch points of a pathway (Sweetlove and Fernie 2018). Moreover, the clear importance of phenylpropanoids in response to stress is underlined by evidence of their antioxidant roles in response to light and water stress (Nakabayashi et al. 2014; Tohge et al. 2016). Intriguingly, the assembly of enzymes of dhurrin metabolism is postulated to be dynamic to cope with increased demand in response to environmental stresses (Bassard et al. 2017).

It is important to note that phase separation has only been formally demonstrated for a small subset of enzyme assemblies (Wunder and Mueller-Cajar 2020); however, by analogy to yeast and mammalian they likely exist. Yeast and mammalian glycolytic enzymes condense into so-called G-bodies during hypoxic stress, and, like many other biomolecular condensates, these G-bodies are enriched in RNA-binding domains and mRNA (Jin et al. 2017; Kohnhorst et al. 2017; Fuller et al. 2020). Furthermore, G-body formation correlates with increased rates of glycolysis (Jin et al. 2017), although whether metabolon formation underlines this increase remains to be resolved. Although these findings are intriguing, further studies are needed to determine if the same mechanisms operate in plants. This caveat notwithstanding, the above-discussed enzyme-enzyme assemblies, beyond the fact that they all contain well-characterized substrate channels, are likely responsive to either biotic or abiotic stresses. Indeed, previous reviews have pondered the different selective pressures that variously brought about dynamic enzyme–enzyme assemblies and stable multi-enzyme complexes (Sweetlove and Fernie 2018). The fact that (dis)assembly of such complexes in response to stress provides a flexible and energetically spartan route to adjust metabolic fluxes in response to stress is certainly a highly feasible reason for the evolution of such dynamic aggregates.

### Stress-induced small molecules in biomolecular condensates

Considering the chemical and functional diversity of small molecules, it is not surprising that they have emerged as components and regulators of biomolecular condensates in plants and animals (Kosmacz et al. 2019; Klein et al. 2020). A recent study reported the presence of dozens of known metabolites sequestered within Arabidopsis cytosolic SGs, including amino acids, nucleotides, and phospholipids (Kosmacz et al. 2019). What drives the sequestration of the different compounds and what their functions are remains to be examined. For instance, it was speculated that proline, known for its chaperoning activities and found in both cytosolic and plastidial SGs, might contribute to the proper folding of proteins sequestered into biomolecular condensates (Dandage et al. 2015; Kosmacz et al. 2019; Chodasiewicz et al. 2020). A different metabolite reproducibly detected in SGs and indispensable for SG dynamics and function is ATP. ATP fuels the activity of RNA and protein chaperone complexes, which are essential components of the SGs in mammalian and yeast cells (Jain et al. 2016; Tauber et al. 2020). Moreover, ATP is a hydrotrope that counters the formation and can also dissolve already formed protein aggregates (Patel et al. 2017). Treatments that dysregulate ATP levels interfere with SG assembly and impede SG disassembly once they are formed (Jain et al. 2016). We are confident that, despite still being in its infancy (especially in plants), the identification and functional characterization of small molecules in biomolecular condensates will provide insight into condensate formation, dynamics, and behavior.

As already discussed, individual enzymes or entire metabolons can localize to biomolecular condensates, which might regulate (stimulate or inhibit) specific biochemical reactions (Fig. 5, C and D). This influence provides a direct link between metabolism, metabolite levels, and biomolecular condensates. One illustrative example with a direct relevance to stress responses comes from yeast. Using an elegant combination of genetics and cell biology, Cereghetti and colleagues showed that SGs are involved in fine-tuning ATP levels (Cereghetti et al. 2021). In glucose-grown yeast cultures, stress inhibits glycolysis, leading to a decrease in fructose 1,6 bisphosphate (FBP) levels. FBP is an allosteric ligand of a pyruvate kinase (cdc19), a glycolytic enzyme lying behind the final, ATP-producing step of the glycolytic pathway. FBP binding to cdc19 promotes its active tetrameric structure. The decrease in FBP concentration results in tetramer disassembly. Monomeric cdc19 is sequestered within SGs, where it is kept inactive. When the stress abates, FBP level rises, and FBP binding to cdc19 promotes recruitment of chaperones and cdc19 resolubilization. In turn, once released from SGs, cdc19 contributes to the increase in the ATP levels required for SG disassembly.

Numerous stress-induced molecules regulate condensate dynamics without necessarily being a condensate component themselves. An example is 2′,3′-cAMP, which is an evolutionarily conserved RNA degradation product known to accumulate in response to stress and injury (Jackson 2017; Londono Velez et al. 2022; Yu et al. 2022). 2′,3′-cAMP treatment of Arabidopsis seedlings affected the levels of hundreds of transcripts, proteins, and metabolites, many of which were previously associated with plant stress responses. In addition, 2′,3′-cAMP–induced SGs affected the motility of PBs (Kosmacz et al. 2018; Chodasiewicz et al. 2022). Although no evidence of 2′,3′-cAMP being sequestered within SGs is available, 2′,3′-cAMP can bind to the RNA-binding motif present in SG core proteins such as RBP47 (Kosmacz et al. 2018). Like 2′,3′-cAMP, SA is also a stress-related small molecule with an ability to promote protein condensation in plants. As described above, SA induces the condensation of cytoplasmic NPR1 and GBPL defense-activated condensates in the nucleus of Arabidopsis plants (Zavaliev et al. 2020; Huang et al. 2021; Kim et al. 2022).

Another example of a metabolite shown to affect the dynamics and function of biomolecular condensates is S-adenosylmethionine (AdoMet). Using a combination of mutants affected in AdoMet metabolism and AdoMet supplementation experiments in yeast and human cell lines, AdoMet was demonstrated to suppress SG formation in response to acute stress and also affect the expression and recruitment of specific SG components (Begovich et al. 2020). AdoMet is a co-substrate involved in methyl group transfer. Although protein methylation does not appear to affect AdoMet function, AdoMet could theoretically work by altering RNA methylation, a hypothesis that requires testing. Intriguingly, S-adenosylmethionine synthase, an enzyme responsible for AdoMet production, is sequestered within SGs, pointing to the existence of a regulatory loop whereby SG sequestration may contribute to the regulation of cellular AdoMet levels. A final example of a metabolite that regulates condensate formation, in this case PBs, is 5-diphosphoinositol pentakisphosphate (InsP7) (Sahu et al. 2020). InsP7 was shown to inhibit the Nudix Hydrolase 3-dependent decapping of mRNAs and increases PB abundance in human cells, with this effect being environmentally and developmentally regulated.

## Harnessing biomolecular condensates for growing resilient plants

Biomolecular condensates have emerged as key players in human health (Spannl et al. 2019; Alberti and Hyman 2021). Neurodegenerative diseases such as Alzheimer's disease or amyotrophic lateral sclerosis have been linked to defects in the condensation of FUS or other prion-like RBPs (Murakami et al. 2015; Patel et al. 2015). Viruses such as herpes simplex or human immunodeficiency have developed mechanisms counteracting SG formation, thus promoting their replication (Mahboubi and Stochaj 2017). In the context of cancer, SGs are in general advantageous to tumors because they enhance cell survival, metastasis, and tolerance to treatments (Gao et al. 2019). In addition, many condensate-associated proteins aberrantly accumulate in cancer cells (Mahboubi and Stochaj 2017; Spannl et al. 2019), whereas defects in protein condensate turnover have been linked to aging and age-related diseases (Lopez-Otin et al. 2013; Alberti and Hyman 2021). All these biomedicine-relevant findings underscore how biomolecular condensates can control cell fate through multiple and interconnected pathways, ranging from regulation of translation to the modulation of various types of cell death. That is why biomolecular condensates are considered as promising targets to improve therapeutic intervention for several diseases.

To gain insight into the biological role of biomolecular condensates, researchers have traditionally focused their efforts on the characterization of loss-of-function mutants in putative components. Two of the best-studied examples in mammalian systems are G3BP1 and G3BP2, 2 SG proteins about which studies have provided invaluable insight into key aspects of SG biology (Cirillo et al. 2020). For example, an initial study revealed that inhibition of SG formation in G3BP-deficient lines prevented metastasis and limited tumor invasion (Somasekharan et al. 2015). A more recent report demonstrated that inhibition of SG assembly under G3BP deficiency could only occur under arsenite stress, suggesting that the core mechanisms of SG formation may differ depending on the initiation stimulus (Yang et al. 2020). Curiously, G3BP-deficient mutants are the only mutants unable to form SGs in nonplant organisms. To date, no such mutants are available in plants. However, a recent study showed that the Arabidopsis putative orthologs *G3BP-1* and *G3BP-7* were able to rescue SG formation when expressed in human cells lacking native G3BP function, pointing to some degree of conservation of SG-forming mechanisms across kingdoms (Reuper et al. 2021). However, the implication of Arabidopsis G3BP proteins in SG assembly has not been fully addressed. Although studies of loss-of-function mutants aid in better understanding biomolecular condensation, new complementary approaches have recently emerged. For example, an automated cell-based assay platform was used for the identification of new molecules affecting PB assembly and provided an important insight into the relationship between PB assembly and diverse intracellular programs, including organelle physiology (Martínez et al. 2013). In line with this notion, a more recent study using a high-content screen identified small molecules that affect SG assembly and modulate inflammatory signaling pathways (Fang et al. 2019a).

In contrast to the emerging role of biomolecular condensates in human diseases, stress-induced condensates such as SGs, PBs, or siRNA bodies have so far received limited attention in plants. Nonetheless, there is growing evidence for a direct link between protein condensation and plant stress tolerance (Jang et al. 2020; Londono Velez et al. 2022). One example is increased freezing tolerance of Arabidopsis plants with a mutation in the mRNA decapping activator SM-like protein LSM1-7 that results in fewer PBs (Perea-Resa et al. 2016). A more recent study showed that the SG component Multiprotein-bridging factor 1c from wheat (*Triticum aestivum*) contributed to the heat tolerance of the plant by regulating heat stress-induced mRNA translation (Tian et al. 2022). Biomedical research suggests that changes in the phase separation behavior of condensate components can induce the formation of aberrant condensates, resulting in diseases (Alberti and Hyman 2021). Although LLPS and other phase separation mechanisms are poorly understood in plants, a recent study of the heat-induced phase separation of Arabidopsis RBGD2 and RBGD4 has provided a strong argument for the importance of phase separation in plant tolerance to heat stress (Zhu et al. 2022). We therefore stand at the very beginning of the exciting path to translate basic knowledge about biomolecular condensates and phase separation to the production of resilient crops.

## Conclusions/outstanding questions

Despite significant progress achieved in the past decade in studying biomolecular condensates in plants, there are still many open questions. Further delving into the compositional and structural complexity of various types of condensates would provide a better clue to the origin of their heterogeneity. Considering the dynamic interaction among different stress-related condensates, including PBs, SGs, and siRNA bodies, one always wonders what is inside one condensate and not in the other at a particular moment of time. Yet, the hottest questions are the following: what are the key players of, and can we abolish condensate formation? To date, most of the research on biomolecular condensates in plants has been performed using one or a few condensate-localized proteins (often fused to a fluorescent reporter protein) that can be observed by microscopy. Although genetic or pharmacological manipulations can abolish or alter the microscopic localization of the reporter, this would not necessarily mean that condensate assembly is abolished or altered as well. Indeed, backup or auxiliary pathways (e.g. through recruitment of alternative scaffolding factors) might be activated and achieve condensate assembly but for some reason without recruiting the reporter under study. A deeper understanding of the mechanisms leading to condensate formation through combination of in vivo and in vitro (e.g. condensate reconstitution) approaches would allow better control over biomolecular condensation in plants, also in the context of stress responses.

## Materials and methods

### Bioinformatics

To retrieve protein–protein interaction data, the STRING database (V10) was used (Szklarczyk et al. 2015). Only physical protein–protein interactions were considered. The RNA-binding proteins were predicted by the RNApred tool (Kumar et al. 2011). The prediction approach was based on amino acid composition, and the threshold for the support vector machine was 0.5. The orthology analysis was performed using eggNOG database (Huerta-Cepas et al. 2019).

## Supplementary Material

koad127_Supplementary_Data

## Supplementary data

The following materials are available in the online version of this article.

**Supplemental Data Set 1.** Full list of SG-associated proteins isolated from TSN2, RBP47, RBGD2/4, and CML38 immunoprecipitations.

**Supplemental Data Set 2.** Full list of PB-associated proteins isolated from DCP1, DCP2, DCP5, and UPF1 immunoprecipitations.

## References

[koad127-B1] Abyzov A , Blackledge M, Zweckstetter M. Conformational dynamics of intrinsically disordered proteins regulate biomolecular condensate chemistry. Chem Rev. 2022:122(6):6719–6748. 10.1021/acs.chemrev.1c00774 35179885 PMC8949871

[koad127-B2] Achnine L , Huhman DV, Farag MA, Sumner LW, Blount JW, Dixon RA. Genomics-based selection and functional characterization of triterpene glycosyltransferases from the model legume Medicago truncatula. Plant J. 2005:41(6):875–887. 10.1111/j.1365-313X.2005.02344.x 15743451

[koad127-B3] Alberti S , Hyman AA. Are aberrant phase transitions a driver of cellular aging? Bioessays. 2016:38(10):959–968. 10.1002/bies.201600042 27554449 PMC5108435

[koad127-B4] Alberti S , Hyman AA. Biomolecular condensates at the nexus of cellular stress, protein aggregation disease and ageing. Nat Rev Mol Cell Biol. 2021:22(3):196–213. 10.1038/s41580-020-00326-6 33510441

[koad127-B5] Ayache J , Bénard M, Ernoult-Lange M, Minshall N, Standart N, Kress M, Weil D. P-body assembly requires DDX6 repression complexes rather than decay or ataxin2/2L complexes. Mol Biol Cell. 2015:26(14):2579–2595. 10.1091/mbc.E15-03-0136 25995375 PMC4501357

[koad127-B6] Banani SF , Lee HO, Hyman AA, Rosen MK. Biomolecular condensates: organizers of cellular biochemistry. Nat Rev Mol Cell Biol. 2017:18(5):285–298. 10.1038/nrm.2017.7 28225081 PMC7434221

[koad127-B7] Bassard JE , Moller BL, Laursen T. Assembly of dynamic P450-mediated metabolons—order versus chaos. Curr Mol Biol Rep. 2017:3(1):37–51. 10.1007/s40610-017-0053-y 28255532 PMC5310546

[koad127-B8] Begovich K , Vu AQ, Yeo G, Wilhelm JE. Conserved metabolite regulation of stress granule assembly via AdoMet. J Cell Biol. 2020:219(8). 10.1083/jcb.201904141 PMC7401819 32609300

[koad127-B9] Bergeron-Sandoval L-P , Michnick SW. Mechanics, structure and function of biopolymer condensates. J Mol Biol. 2018:430(23):4754–4761. 10.1016/j.jmb.2018.06.023 29913159

[koad127-B10] Brangwynne CP , Eckmann CR, Courson DS, Rybarska A, Hoege C, Gharakhani J, Jülicher F, Hyman AA. Germline P granules are liquid droplets that localize by controlled dissolution/condensation. Science. 2009:324(5935):1729–1732. 10.1126/science.1172046 19460965

[koad127-B11] Burbulis IE , Winkel-Shirley B. Interactions among enzymes of the *Arabidopsis* flavonoid biosynthetic pathway. Proc Natl Acad Sci USA. 1999:96(22):12929–12934. 10.1073/pnas.96.22.12929 10536025 PMC23169

[koad127-B12] Case LB . Membranes regulate biomolecular condensates. Nat Cell Biol. 2022:24(4):404–405. 10.1038/s41556-022-00892-1 35411084

[koad127-B13] Cereghetti G , Wilson-Zbinden C, Kissling VM, Diether M, Arm A, Yoo H, Piazza I, Saad S, Picotti P, Drummond DA, et al Reversible amyloids of pyruvate kinase couple cell metabolism and stress granule disassembly. Nat Cell Biol. 2021:23(10):1085–1094. 10.1038/s41556-021-00760-4 34616026 PMC7611853

[koad127-B14] Chantarachot T , Sorenson RS, Hummel M, Ke H, Kettenburg AT, Chen D, Aiyetiwa K, Dehesh K, Eulgem T, Sieburth LE, et al DHH1/DDX6-like RNA helicases maintain ephemeral half-lives of stress-response mRNAs. Nat Plants. 2020:6(6):675–685. 10.1038/s41477-020-0681-8 32483330

[koad127-B15] Chicois C , Scheer H, Garcia S, Zuber H, Mutterer J, Chicher J, Hammann P, Gagliardi D, Garcia D. The UPF1 interactome reveals interaction networks between RNA degradation and translation repression factors in Arabidopsis. Plant J. 2018:96(1):119–132. 10.1111/tpj.14022 29983000

[koad127-B16] Chodasiewicz M , Kerber O, Gorka M, Moreno JC, Maruri-Lopez I, Minen RI, Sampathkumar A, Nelson ADL, Skirycz A. 2′,3′-cAMP Treatment mimics the stress molecular response in *Arabidopsis thaliana*. Plant Physiol. 2022:188(4):1966–1978. 10.1093/plphys/kiac013 35043968 PMC8968299

[koad127-B17] Chodasiewicz M , Sokolowska EM, Nelson-Dittrich AC, Masiuk A, Beltran JCM, Nelson ADL, Skirycz A. Identification and characterization of the heat-induced plastidial stress granules reveal new insight into *Arabidopsis* stress response. Front Plant Sci. 2020:11:595792. 10.3389/fpls.2020.595792 PMC7674640 33224174

[koad127-B18] Chung WC , Ahn JH, Song MJ. Liquid-liquid phase separation drives herpesvirus assembly in the cytoplasm. J Cell Biol. 2023:222(1). 10.1083/jcb.202211015 PMC9779906 36542408

[koad127-B19] Cirillo L , Cieren A, Barbieri S, Khong A, Schwager F, Parker R, Gotta M. UBAP2L Forms distinct cores that act in nucleating stress granules upstream of G3BP1. Curr Biol. 2020:30(4):698–707.e6. 10.1016/j.cub.2019.12.020 31956030

[koad127-B20] Cohan MC , Pappu RV. Making the case for disordered proteins and biomolecular condensates in Bacteria. Trends Biochem Sci. 2020:45(8):668–680. 10.1016/j.tibs.2020.04.011 32456986

[koad127-B21] Conti BA , Oppikofer M. Biomolecular condensates: new opportunities for drug discovery and RNA therapeutics. Trends Pharmacol Sci. 2022:43(10):820–837. 10.1016/j.tips.2022.07.001 36028355

[koad127-B22] Crosby KC , Pietraszewska-Bogiel A, Gadella TW Jr, Winkel BS. Förster resonance energy transfer demonstrates a flavonoid metabolon in living plant cells that displays competitive interactions between enzymes. FEBS Lett. 2011:585(14):2193–2198. 10.1016/j.febslet.2011.05.066 21669202

[koad127-B23] Dandage R , Bandyopadhyay A, Jayaraj GG, Saxena K, Dalal V, Das A, Chakraborty K. Classification of chemical chaperones based on their effect on protein folding landscapes. ACS Chem Biol. 2015:10(3):813–820. 10.1021/cb500798y 25493352

[koad127-B24] Dine E , Gil AA, Uribe G, Brangwynne CP, Toettcher JE. Protein phase separation provides long-term memory of transient spatial stimuli. Cell Syst. 2018:6(6):655–663.e5. 10.1016/j.cels.2018.05.002 29859829 PMC6023754

[koad127-B25] Di Stefano B , Luo E-C, Haggerty C, Aigner S, Charlton J, Brumbaugh J, Ji F, Rabano Jiménez I, Clowers KJ, Huebner AJ, et al The RNA helicase DDX6 controls cellular plasticity by modulating P-body homeostasis. Cell Stem Cell. 2019:25(5):622–638.e13. 10.1016/j.stem.2019.08.018 31588046 PMC7247364

[koad127-B26] Ditlev JA , Case LB, Rosen MK. Who's in and who's out—compositional control of biomolecular condensates. J Mol Biol. 2018:430(23):4666–4684. 10.1016/j.jmb.2018.08.003 30099028 PMC6204295

[koad127-B27] Dorone Y , Boeynaems S, Flores E, Jin B, Hateley S, Bossi F, Lazarus E, Pennington JG, Michiels E, De Decker M, et al A prion-like protein regulator of seed germination undergoes hydration-dependent phase separation. Cell. 2021:184(16):4284–4298.e27. 10.1016/j.cell.2021.06.009 34233164 PMC8513799

[koad127-B28] Emenecker RJ , Holehouse AS, Strader LC. Emerging roles for phase separation in plants. Dev Cell. 2020:55(1):69–83. 10.1016/j.devcel.2020.09.010 33049212 PMC7577370

[koad127-B29] Fang MY , Markmiller S, Vu AQ, Javaherian A, Dowdle WE, Jolivet P, Bushway PJ, Castello NA, Baral A, Chan MY, et al Small-Molecule modulation of TDP-43 recruitment to stress granules prevents persistent TDP-43 accumulation in ALS/FTD. Neuron. 2019a:103(5):802–819.e11. 10.1016/j.neuron.2019.05.048 31272829 PMC6728177

[koad127-B30] Fang X , Wang L, Ishikawa R, Li Y, Fiedler M, Liu F, Calder G, Rowan B, Weigel D, Li P, et al Arabidopsis FLL2 promotes liquid-liquid phase separation of polyadenylation complexes. Nature. 2019b:569(7755):265–269. 10.1038/s41586-019-1165-8 31043738 PMC6625965

[koad127-B31] Fare CM , Villani A, Drake LE, Shorter J. Higher-order organization of biomolecular condensates. Open Biol. 2021:11(6):210137. 10.1098/rsob.210137 PMC8205532 34129784

[koad127-B32] Field S , Conner WC, Roberts DM. Arabidopsis CALMODULIN-LIKE 38 regulates hypoxia-induced autophagy of SUPPRESSOR OF GENE SILENCING 3 bodies. Front Plant Sci. 2021:12:722940. 10.3389/fpls.2021.722940 PMC8456008 34567037

[koad127-B33] Freeman Rosenzweig ES , Xu B, Kuhn Cuellar L, Martinez-Sanchez A, Schaffer M, Strauss M, Cartwright HN, Ronceray P, Plitzko JM, Förster F, et al The eukaryotic CO(2)-concentrating organelle is liquid-like and exhibits dynamic reorganization. Cell. 2017:171(1):148–162.e19. 10.1016/j.cell.2017.08.008 28938114 PMC5671343

[koad127-B34] Fuller GG , Han T, Freeberg MA, Moresco JJ, Ghanbari Niaki A, Roach NP, Yates JR, Myong S, Kim JK. RNA Promotes phase separation of glycolysis enzymes into yeast G bodies in hypoxia. Elife. 2020:9. 10.7554/eLife.48480 PMC7162659 32298230

[koad127-B35] Gao X , Jiang L, Gong Y, Chen X, Ying M, Zhu H, He Q, Yang B, Cao J. Stress granule: a promising target for cancer treatment. Br J Pharmacol. 2019:176(23):4421–4433. 10.1111/bph.14790 31301065 PMC6932939

[koad127-B36] Gao Y , Zhu Y, Wang H, Cheng Y, Zhao D, Sun Q, Chen D. Lipid-mediated phase separation of AGO proteins on the ER controls nascent-peptide ubiquitination. Mol Cell. 2022:82(7):1313–1328.e8. 10.1016/j.molcel.2022.02.035 35325613

[koad127-B37] Garaizar A , Espinosa JR, Joseph JA, Collepardo-Guevara R. Kinetic interplay between droplet maturation and coalescence modulates shape of aged protein condensates. Sci Rep. 2022:12(1):4390. 10.1038/s41598-022-08130-2 35293386 PMC8924231

[koad127-B38] Glauninger H , Wong Hickernell CJ, Bard JAM, Drummond DA. Stressful steps: progress and challenges in understanding stress-induced mRNA condensation and accumulation in stress granules. Mol Cell. 2022:82(14):2544–2556. 10.1016/j.molcel.2022.05.014 35662398 PMC9308734

[koad127-B39] Gomes E , Shorter J. The molecular language of membraneless organelles. J Biol Chem. 2019:294(18):7115–7127. 10.1074/jbc.TM118.001192 30045872 PMC6509512

[koad127-B40] Gutierrez-Beltran E , Elander PH, Dalman K, Dayhoff GW, Moschou PN, Uversky VN, Crespo JL, Bozhkov PV. Tudor staphylococcal nuclease is a docking platform for stress granule components and is essential for SnRK1 activation in *Arabidopsis*. EMBO J. 2021:40(17):e105043. 10.15252/embj.2020105043 PMC8447601 34287990

[koad127-B41] Gutierrez-Beltran E , Moschou PN, Smertenko AP, Bozhkov PV. Tudor staphylococcal nuclease links formation of stress granules and processing bodies with mRNA catabolism in *Arabidopsis*. Plant Cell. 2015:27(3):926–943. 10.1105/tpc.114.134494 25736060 PMC4558657

[koad127-B42] Han T , Kato M, Xie S, Wu L, Mirzaei H, Pei J, Chen M, Xie Y, Allen J, Xiao G, et al Cell-free formation of RNA granules: bound RNAs identify features and components of cellular assemblies. Cell. 2012:149(4):768–779. 10.1016/j.cell.2012.04.016 22579282

[koad127-B43] Hardenberg MC , Sinnige T, Casford S, Dada ST, Poudel C, Robinson EA, Fuxreiter M, Kaminksi CF, Kaminski Schierle GS, Nollen EA, et al Observation of an alpha-synuclein liquid droplet state and its maturation into Lewy body-like assemblies. J Mol Cell Biol. 2021:13:282–294. 10.1093/jmcb/mjaa075 33386842 PMC8339365

[koad127-B44] Hoffmann G , Mahboubi A, Bente H, Garcia D, Hanson J, Hafren A. Arabidopsis RNA processing body components LSM1 and DCP5 aid in the evasion of translational repression during *Cauliflower mosaic virus* infection. Plant Cell. 2022:34(8):3128–3147. 10.1093/plcell/koac132 35511183 PMC9338796

[koad127-B45] Hou Q , Ufer G, Bartels D. Lipid signalling in plant responses to abiotic stress. Plant Cell Environ. 2016:39(5):1029–1048. 10.1111/pce.12666 26510494

[koad127-B46] Huang S , Zhu S, Kumar P, MacMicking JD. A phase-separated nuclear GBPL circuit controls immunity in plants. Nature. 2021:594(7863):424–429. 10.1038/s41586-021-03572-6 34040255 PMC8478157

[koad127-B47] Hubstenberger A , Courel M, Bénard M, Souquere S, Ernoult-Lange M, Chouaib R, Yi Z, Morlot JB, Munier A, Fradet M, et al P-Body purification reveals the condensation of repressed mRNA regulons. Mol Cell. 2017:68(1):144–157.e5. 10.1016/j.molcel.2017.09.003 28965817

[koad127-B48] Huerta-Cepas J , Szklarczyk D, Heller D, Hernández-Plaza A, Forslund SK, Cook H, Mende DR, Letunic I, Rattei T, Jensen L, et al eggNOG 5.0: a hierarchical, functionally and phylogenetically annotated orthology resource based on 5090 organisms and 2502 viruses. Nucleic Acids Res. 2019:47(D1):D309–D314. 10.1093/nar/gky1085 30418610 PMC6324079

[koad127-B49] Iserman C , Desroches Altamirano C, Jegers C, Friedrich U, Zarin T, Fritsch AW, Mittasch M, Domingues A, Hersemann L, Jahnel M, et al Condensation of Ded1p promotes a translational switch from housekeeping to stress protein production. Cell. 2020:181(4):818–831.e19. 10.1016/j.cell.2020.04.009 32359423 PMC7237889

[koad127-B50] Jackson EK . Discovery and roles of 2′,3′-cAMP in biological systems. Handb Exp Pharmacol. 2017:238:229–252. 10.1007/164_2015_40 26721674

[koad127-B51] Jain S , Wheeler JR, Walters RW, Agrawal A, Barsic A, Parker R. ATPase-Modulated stress granules contain a diverse proteome and substructure. Cell. 2016:164(3):487–498. 10.1016/j.cell.2015.12.038 26777405 PMC4733397

[koad127-B52] Jang GJ , Jang JC, Wu SH. Dynamics and functions of stress granules and processing bodies in plants. Plants (Basel). 2020:9(9):1122. 10.3390/plants9091122 32872650 PMC7570210

[koad127-B53] Jin M , Fuller GG, Han T, Yao Y, Alessi AF, Freeberg MA, Roach NP, Moresco JJ, Karnovsky A, Baba M, et al Glycolytic enzymes coalesce in G bodies under hypoxic stress. Cell Rep. 2017:20(4):895–908. 10.1016/j.celrep.2017.06.082 28746874 PMC5586494

[koad127-B54] Jouannet V , Moreno AB, Elmayan T, Vaucheret H, Crespi MD, Maizel A. Cytoplasmic *Arabidopsis* AGO7 accumulates in membrane-associated siRNA bodies and is required for ta-siRNA biogenesis. EMBO J. 2012:31(7):1704–1713. 10.1038/emboj.2012.20 22327216 PMC3321200

[koad127-B55] Kearly A , Nelson ADL, Skirycz A, Chodasiewicz M. Composition and function of stress granules and P-bodies in plants. Semin Cell Dev Biol. 2022:S1084-9521(22)00350-0. 10.1016/j.semcdb.2022.11.008 36464613

[koad127-B56] Kerfeld CA , Aussignargues C, Zarzycki J, Cai F, Sutter M. Bacterial microcompartments. Nature Reviews Microbiology. 2018:16(5):277–290. 10.1038/nrmicro.2018.10 29503457 PMC6022854

[koad127-B58] Kim EY , Wang L, Lei Z, Li H, Fan W, Cho J. Ribosome stalling and SGS3 phase separation prime the epigenetic silencing of transposons. Nat Plants. 2021:7(3):303–309. 10.1038/s41477-021-00867-4 33649597

[koad127-B57] Kim JH , Castroverde CDM, Huang S, Li C, Hilleary R, Seroka A, Sohrabi R, Medina-Yerena D, Huot B, Wang J, et al Increasing the resilience of plant immunity to a warming climate. Nature. 2022:607(7918):339–344. 10.1038/s41586-022-04902-y 35768511 PMC9279160

[koad127-B59] Klein IA , Boija A, Afeyan LK, Hawken SW, Fan M, Dall'Agnese A, Oksuz O, Henninger JE, Shrinivas K, Sabari BR, et al Partitioning of cancer therapeutics in nuclear condensates. Science. 2020:368(6497):1386–1392. 10.1126/science.aaz4427 32554597 PMC7735713

[koad127-B60] Kohnhorst CL , Kyoung M, Jeon M, Schmitt DL, Kennedy EL, Ramirez J, Bracey SM, Luu BT, Russell SJ, An S. Identification of a multienzyme complex for glucose metabolism in living cells. J Biol Chem. 2017:292(22):9191–9203. 10.1074/jbc.M117.783050 28424264 PMC5454101

[koad127-B61] Kosmacz M , Gorka M, Schmidt S, Luzarowski M, Moreno JC, Szlachetko J, Leniak E, Sokolowska EM, Sofroni K, Schnittger A, et al Protein and metabolite composition of *Arabidopsis* stress granules. New Phytol. 2019:222(3):1420–1433. 10.1111/nph.15690 30664249

[koad127-B62] Kosmacz M , Luzarowski M, Kerber O, Leniak E, Gutiérrez-Beltrán E, Moreno JC, Gorka M, Szlachetko J, Veyel D, Graf A, et al Interaction of 2′,3′-cAMP with Rbp47b plays a role in stress granule formation. Plant Physiol. 2018:177:411–421. 10.1104/pp.18.00285 29618637 PMC5933139

[koad127-B63] Krainer G , Welsh TJ, Joseph JA, Espinosa JR, Wittmann S, de Csilléry E, Sridhar A, Toprakcioglu Z, Gudiškytė G, Czekalska MA, et al Reentrant liquid condensate phase of proteins is stabilized by hydrophobic and non-ionic interactions. Nat Commun. 2021:12(1):1085. 10.1038/s41467-021-21181-9 33597515 PMC7889641

[koad127-B64] Kumar M , Gromiha MM, Raghava GP. SVM based prediction of RNA-binding proteins using binding residues and evolutionary information. J Mol Recognit. 2011:24(2):303–313. 10.1002/jmr.1061 20677174

[koad127-B65] Kusumaatmaja H , May AI, Knorr RL. Intracellular wetting mediates contacts between liquid compartments and membrane-bound organelles. J Cell Biol. 2021:220(10):e202103175. 10.1083/jcb.202103175 34427635 PMC8404468

[koad127-B67] Li HR , Chiang WC, Chou PC, Wang WJ, Huang JR. TAR DNA-binding protein 43 (TDP-43) liquid-liquid phase separation is mediated by just a few aromatic residues. J Biol Chem. 2018:293(16):6090–6098. 10.1074/jbc.AC117.001037 29511089 PMC5912450

[koad127-B68] Li F , Zhao N, Li Z, Xu X, Wang Y, Yang X, Liu S-S, Wang A, Zhou X. A calmodulin-like protein suppresses RNA silencing and promotes geminivirus infection by degrading SGS3 via the autophagy pathway in Nicotiana benthamiana. PLoS Pathog. 2017:13(2):e1006213. 10.1371/journal.ppat.1006213 PMC5333915 28212430

[koad127-B66] Li P , Banjade S, Cheng H-C, Kim S, Chen B, Guo L, Llaguno M, Hollingsworth JV, King DS, Banani SF, et al Phase transitions in the assembly of multivalent signalling proteins. Nature. 2012:483(7389):336–340. 10.1038/nature10879 22398450 PMC3343696

[koad127-B69] Liu C , Mentzelopoulou A, Deli A, Papagavriil F, Ramachandran P, Perraki A, Claus L, Barg S, Dörmann P, Jaillais Y, et al Phase transitions of a SEC14-like condensate at Arabidopsis plasma membranes regulate root growth. bioRxiv. 2022. 10.1371/journal.pbio.3002305 PMC10538751 37721949

[koad127-B70] Liu C , Mentzelopoulou A, Muhammad A, Volkov A, Weijers D, Gutierrez-Beltran E, Moschou PN. An actin remodeling role for *Arabidopsis* processing bodies revealed by their proximity interactome. EMBO J. 2023:42(9):e111885. 10.15252/embj.2022111885 36741000 PMC10152145

[koad127-B71] Lokdarshi A , Conner WC, McClintock C, Li T, Roberts D. Arabidopsis CML38, a calcium sensor that localizes to ribonucleoprotein complexes under hypoxia stress. Plant Physiol. 2015:170(2):1046–1059. 10.1104/pp.15.01407 26634999 PMC4734562

[koad127-B72] Londono Velez V , Alquraish F, Tarbiyyah I, Rafique F, Mao D, Chodasiewicz M. Landscape of biomolecular condensates in heat stress responses. Front Plant Sci. 2022:13:1032045. 10.3389/fpls.2022.1032045 PMC9601738 36311142

[koad127-B73] Lopez-Otin C , Blasco MA, Partridge L, Serrano M, Kroemer G. The hallmarks of aging. Cell. 2013:153(6):1194–1217. 10.1016/j.cell.2013.05.039 23746838 PMC3836174

[koad127-B74] Luo Y , Na Z, Slavoff SA. P-Bodies: composition, properties, and functions. Biochemistry. 2018:57(17):2424–2431. 10.1021/acs.biochem.7b01162 29381060 PMC6296482

[koad127-B75] Mahboubi H , Stochaj U. Cytoplasmic stress granules: dynamic modulators of cell signaling and disease. Biochim Biophys Acta. 2017:1863(4):884–895. 10.1016/j.bbadis.2016.12.022 28095315

[koad127-B76] Makinen K , Lohmus A, Pollari M. Plant RNA regulatory network and RNA granules in virus infection. Front Plant Sci. 2017:8:2093. 10.3389/fpls.2017.02093 29312371 PMC5732267

[koad127-B77] Markmiller S , Soltanieh S, Server KL, Mak R, Jin W, Fang MY, Luo E-C, Krach F, Yang D, Sen A, et al Context-Dependent and disease-specific diversity in protein interactions within stress granules. Cell. 2018:172(3):590–604.e13. 10.1016/j.cell.2017.12.032 29373831 PMC5969999

[koad127-B78] Marmor-Kollet H , Siany A, Kedersha N, Knafo N, Rivkin N, Danino YM, Moens TG, Olender T, Sheban D, Cohen N, et al Spatiotemporal proteomic analysis of stress granule disassembly using APEX reveals regulation by SUMOylation and links to ALS pathogenesis. Mol Cell. 2020:80(5):876–891.e6. 10.1016/j.molcel.2020.10.032 33217318 PMC7816607

[koad127-B80] Martínez JP , Pérez-Vilaró G, Muthukumar Y, Scheller N, Hirsch T, Diestel R, Steinmetz H, Jansen R, Frank R, Sasse F, et al Screening of small molecules affecting mammalian P-body assembly uncovers links with diverse intracellular processes and organelle physiology. RNA Biol. 2013:10(11):1661–1669. 10.4161/rna.26851 24418890 PMC3907476

[koad127-B81] Martínez de Alba AE , Moreno AB, Gabriel M, Mallory AC, Christ A, Bounon R, Balzergue S, Aubourg S, Gautheret D, Crespi MD, et al In plants, decapping prevents RDR6-dependent production of small interfering RNAs from endogenous mRNAs. Nucleic Acids Res. 2015:43(5):2902–2913. 10.1093/nar/gkv119 25694514 PMC4357720

[koad127-B79] Martinez-Perez M , Aparicio F, Lopez-Gresa MP, Belles JM, Sanchez-Navarro JA, Pallas V. *Arabidopsis* m^6^A demethylase activity modulates viral infection of a plant virus and the m^6^A abundance in its genomic RNAs. Proc Natl Acad Sci U S A. 2017:114(40):10755–10760. 10.1073/pnas.1703139114 28923956 PMC5635872

[koad127-B82] Maruri-Lopez I , Figueroa NE, Hernandez-Sanchez IE, Chodasiewicz M. Plant stress granules: trends and beyond. Front Plant Sci. 2021:12:722643. 10.3389/fpls.2021.722643 PMC8381727 34434210

[koad127-B83] Mateju D , Eichenberger B, Voigt F, Eglinger J, Roth G, Chao JA. Single-Molecule imaging reveals translation of mRNAs localized to stress granules. Cell. 2020:183(7):1801–1812.e13. 10.1016/j.cell.2020.11.010 33308477

[koad127-B84] Meyer EA , Castellano RK, Diederich F. Interactions with aromatic rings in chemical and biological recognition. Angew Chem Int Ed Engl. 2003:42(11):1210–1250. 10.1002/anie.200390319 12645054

[koad127-B85] Millar SR , Huang JQ, Schreiber KJ, Tsai YC, Won J, Zhang J, Moses AM, Youn JY. A new phase of networking: the molecular composition and regulatory dynamics of mammalian stress granules. Chem Rev. 2023. 10.1021/acs.chemrev.2c00608 PMC10375481 36662637

[koad127-B86] Mitrea DM , Mittasch M, Gomes BF, Klein IA, Murcko MA. Modulating biomolecular condensates: a novel approach to drug discovery. Nat Rev Drug Discov. 2022:21(11):841–862. 10.1038/s41573-022-00505-4 35974095 PMC9380678

[koad127-B87] Mittag T , Parker R. Multiple modes of protein-protein interactions promote RNP granule assembly. J Mol Biol. 2018:430(23):4636–4649. 10.1016/j.jmb.2018.08.005 30099026 PMC6204294

[koad127-B88] Mountourakis F , Hatzianestis IH, Stavridou S, Bozhkov PV, Moschou PN. Concentrating and sequestering biomolecules in condensates: impact on plant biology. J Exp Bot. 2023:74(5):1303–1308. 10.1093/jxb/erac497 36516452 PMC10010603

[koad127-B89] Murakami T , Qamar S, Lin J, Schierle G, Rees E, Miyashita A, Costa A, Dodd R, Chan FS, Michel C, et al ALS/FTD mutation-induced phase transition of FUS liquid droplets and reversible hydrogels into irreversible hydrogels impairs RNP granule function. Neuron. 2015:88(4):678–690. 10.1016/j.neuron.2015.10.030 26526393 PMC4660210

[koad127-B90] Murthy AC , Dignon GL, Kan Y, Zerze GH, Parekh SH, Mittal J, Fawzi NL. Molecular interactions underlying liquid-liquid phase separation of the FUS low-complexity domain. Nat Struct Mol Biol. 2019:26(7):637–648. 10.1038/s41594-019-0250-x 31270472 PMC6613800

[koad127-B91] Musacchio A . On the role of phase separation in the biogenesis of membraneless compartments. EMBO J. 2022:41(5):e109952. 10.15252/embj.2021109952 PMC8886532 35107832

[koad127-B92] Nakabayashi R , Yonekura-Sakakibara K, Urano K, Suzuki M, Yamada Y, Nishizawa T, Matsuda F, Kojima M, Sakakibara H, Shinozaki K, et al Enhancement of oxidative and drought tolerance in *Arabidopsis* by overaccumulation of antioxidant flavonoids. Plant J. 2014:77(3):367–379. 10.1111/tpj.12388 24274116 PMC4282528

[koad127-B93] Nott T , Petsalaki E, Farber P, Jervis D, Fussner E, Plochowietz A, Craggs TD, Bazett-Jones D, Pawson T, Forman-Kay J, et al Phase transition of a disordered nuage protein generates environmentally responsive membraneless organelles. Mol Cell. 2015:57(5):936–947. 10.1016/j.molcel.2015.01.013 25747659 PMC4352761

[koad127-B94] Ouazan-Reboul V , Agudo-Canalejo J, Golestanian R. Non-equilibrium phase separation in mixtures of catalytically active particles: size dispersity and screening effects. Eur Phys J E Soft Matter. 2021:44(9):113. 10.1140/epje/s10189-021-00118-6 34478002 PMC8416889

[koad127-B95] Pappu RV . Phase separation-A physical mechanism for organizing information and biochemical reactions. Dev Cell. 2020:55(1):1–3. 10.1016/j.devcel.2020.09.023 33049210

[koad127-B96] Patel A , Lee H, Jawerth L, Maharana S, Jahnel M, Hein M, Stoynov S, Mahamid J, Saha S, Franzmann T, et al A liquid-to-solid phase transition of the ALS protein FUS accelerated by disease mutation. Cell. 2015:162(5):1066–1077. 10.1016/j.cell.2015.07.047 26317470

[koad127-B97] Patel A , Malinovska L, Saha S, Wang J, Alberti S, Krishnan Y, Hyman AA. ATP as a biological hydrotrope. Science. 2017:356(6339):753–756. 10.1126/science.aaf6846 28522535

[koad127-B98] Pedley AM , Boylan JP, Chan CY, Kennedy EL, Kyoung M, Benkovic SJ. Purine biosynthetic enzymes assemble into liquid-like condensates dependent on the activity of chaperone protein HSP90. J Biol Chem. 2022:298(5):101845. 10.1016/j.jbc.2022.101845 PMC9034097 35307352

[koad127-B99] Peeples W , Rosen MK. Mechanistic dissection of increased enzymatic rate in a phase-separated compartment. Nat Chem Biol. 2021:17(6):693–702. 10.1038/s41589-021-00801-x 34035521 PMC8635274

[koad127-B100] Perea-Resa C , Carrasco-López C, Catalá R, Turečková V, Novak O, Zhang W, Sieburth L, Jiménez-Gómez JM, Salinas J. The LSM1-7 complex differentially regulates *Arabidopsis* tolerance to abiotic stress conditions by promoting selective mRNA decapping. Plant Cell. 2016:28(2):505–520. 10.1105/tpc.15.00867 26764377 PMC4790874

[koad127-B101] Powers SK , Holehouse AS, Korasick DA, Schreiber KH, Clark NM, Jing H, Emenecker R, Han S, Tycksen E, Hwang I, et al Nucleo-cytoplasmic partitioning of ARF proteins controls auxin responses in *Arabidopsis thaliana*. Mol Cell. 2019:76(1):177–190.e5. 10.1016/j.molcel.2019.06.044 31421981 PMC6778021

[koad127-B102] Protter DS , Parker R. Principles and properties of stress granules. Trends Cell Biol. 2016a:26(9):668–679. 10.1016/j.tcb.2016.05.004 27289443 PMC4993645

[koad127-B103] Rasmussen S , Dixon RA. Transgene-mediated and elicitor-induced perturbation of metabolic channeling at the entry point into the phenylpropanoid pathway. Plant Cell. 1999:11(8):1537–1551. 10.1105/tpc.11.8.1537 10449586 PMC144296

[koad127-B104] Reuper H , Götte B, Williams L, Tan TJC, McInerney GM, Panas MD, Krenz B. Arabidopsis thaliana G3BP ortholog rescues mammalian stress granule phenotype across kingdoms. Int J Mol Sci. 2021:22(12):6287. 10.3390/ijms22126287 34208100 PMC8230867

[koad127-B105] Riback JA , Zhu L, Ferrolino MC, Tolbert M, Mitrea DM, Sanders DW, Wei M-T, Kriwacki RW, Brangwynne CP. Composition-dependent thermodynamics of intracellular phase separation. Nature. 2020:581(7807):209–214. 10.1038/s41586-020-2256-2 32405004 PMC7733533

[koad127-B106] Robinson JB Jr , Inman L, Sumegi B, Srere PA. Further characterization of the krebs tricarboxylic acid cycle metabolon. J Biol Chem. 1987:262(4):1786–1790. 10.1016/S0021-9258(19)75707-X 2433288

[koad127-B107] Rogg LE , Bartel B. Auxin signaling: derepression through regulated proteolysis. Dev Cell. 2001:1(5):595–604. 10.1016/S1534-5807(01)00077-6 11709180

[koad127-B108] Sahu S , Wang Z, Jiao X, Gu C, Jork N, Wittwer C, Li X, Hostachy S, Fiedler D, Wang H, et al Insp_7_ is a small-molecule regulator of NUDT3-mediated mRNA decapping and processing-body dynamics. Proc Natl Acad Sci U S A. 2020:117(32):19245–19253. 10.1073/pnas.1922284117 32727897 PMC7431097

[koad127-B109] Sanders DW , Kedersha N, Lee DSW, Strom AR, Drake V, Riback JA, Bracha D, Eeftens JM, Iwanicki A, Wang A, et al Competing protein-RNA interaction networks control multiphase intracellular organization. Cell. 2020:181(2):306–324.e28. 10.1016/j.cell.2020.03.050 32302570 PMC7816278

[koad127-B110] Schiaffini M , Chicois C, Pouclet A, Chartier T, Ubrig E, Gobert A, Zuber H, Mutterer J, Chicher J, Kuhn L, et al A NYN domain protein directly interacts with DECAPPING1 and is required for phyllotactic pattern. Plant Physiol. 2022:188(2):1174–1188. 10.1093/plphys/kiab529 34791434 PMC8825452

[koad127-B111] Schuster BS , Dignon GL, Tang WS, Kelley FM, Ranganath AK, Jahnke CN, Simpkins AG, Regy RM, Hammer DA, Good MC, et al Identifying sequence perturbations to an intrinsically disordered protein that determine its phase-separation behavior. Proc Natl Acad Sci U S A. 2020:117(21):11421–11431. 10.1073/pnas.2000223117 32393642 PMC7261017

[koad127-B112] Schutz S , Noldeke ER, Sprangers R. A synergistic network of interactions promotes the formation of in vitro processing bodies and protects mRNA against decapping. Nucleic Acids Res. 2017:45(11):6911–6922. 10.1093/nar/gkx353 28472520 PMC5499654

[koad127-B113] Shen JR . The structure of photosystem II and the mechanism of water oxidation in photosynthesis. Annu Rev Plant Biol. 2015:66(1):23–48. 10.1146/annurev-arplant-050312-120129 25746448

[koad127-B114] Shen Y , Ruggeri FS, Vigolo D, Kamada A, Qamar S, Levin A, Iserman C, Alberti S, George-Hyslop PS, Knowles TPJ. Biomolecular condensates undergo a generic shear-mediated liquid-to-solid transition. Nat Nanotechnol. 2020:15(10):841–847. 10.1038/s41565-020-0731-4 32661370 PMC7116851

[koad127-B115] Somasekharan SP , El-Naggar A, Leprivier G, Cheng H, Hajee S, Grunewald TGP, Zhang F, Ng T, Delattre O, Evdokimova V, et al YB-1 regulates stress granule formation and tumor progression by translationally activating G3BP1. J Cell Biol. 2015:208(7):913–929. 10.1083/jcb.201411047 25800057 PMC4384734

[koad127-B116] Souquere S , Mollet S, Kress M, Dautry F, Pierron G, Weil D. Unravelling the ultrastructure of stress granules and associated P-bodies in human cells. J Cell Sci. 2009:122(20):3619–3626. 10.1242/jcs.054437 19812307

[koad127-B117] Spannl S , Tereshchenko M, Mastromarco GJ, Ihn SJ, Lee HO. Biomolecular condensates in neurodegeneration and cancer. Traffic. 2019:20(12):890–911. 10.1111/tra.12704 31606941

[koad127-B118] Su X , Ditlev JA, Hui E, Xing W, Banjade S, Okrut J, King DS, Taunton J, Rosen MK, Vale RD. Phase separation of signaling molecules promotes T cell receptor signal transduction. Science. 2016:352(6285):595–599. 10.1126/science.aad9964 27056844 PMC4892427

[koad127-B119] Sweetlove LJ , Fernie AR. The spatial organization of metabolism within the plant cell. Annu Rev Plant Biol. 2013:64(1):723–746. 10.1146/annurev-arplant-050312-120233 23330793

[koad127-B120] Sweetlove LJ , Fernie AR. The role of dynamic enzyme assemblies and substrate channelling in metabolic regulation. Nat Commun. 2018:9(1):2136. 10.1038/s41467-018-04543-8 29849027 PMC5976638

[koad127-B121] Szecowka M , Heise R, Tohge T, Nunes-Nesi A, Vosloh D, Huege J, Feil R, Lunn J, Nikoloski Z, Stitt M, et al Metabolic fluxes in an illuminated *Arabidopsis* rosette. Plant Cell. 2013:25(2):694–714. 10.1105/tpc.112.106989 23444331 PMC3608787

[koad127-B122] Szklarczyk D , Franceschini A, Wyder S, Forslund K, Heller D, Huerta-Cepas J, Simonovic M, Roth A, Santos A, Tsafou KP, et al STRING V10: protein-protein interaction networks, integrated over the tree of life. Nucleic Acids Res. 2015:43(D1):D447–D452. 10.1093/nar/gku1003 25352553 PMC4383874

[koad127-B123] Tauber D , Tauber G, Khong A, Van Treeck B, Pelletier J, Parker R. Modulation of RNA condensation by the DEAD-box protein eIF4A. Cell. 2020:180(3):411–426.e16. 10.1016/j.cell.2019.12.031 31928844 PMC7194247

[koad127-B124] Tian X , Qin Z, Zhao Y, Wen J, Lan T, Zhang L, Wang F, Qin D, Yu K, Zhao A, et al Stress granule-associated TaMBF1c confers thermotolerance through regulating specific mRNA translation in wheat (*Triticum aestivum*). New Phytol. 2022:233(4):1719–1731. 10.1111/nph.17865 34787921 PMC9300156

[koad127-B125] Tohge T , Wendenburg R, Ishihara H, Nakabayashi R, Watanabe M, Sulpice R, Hoefgen R, Takayama H, Saito K, Stitt M, et al Characterization of a recently evolved flavonol-phenylacyltransferase gene provides signatures of natural light selection in Brassicaceae. Nat Commun. 2016:7(1):12399. 10.1038/ncomms12399 27545969 PMC4996938

[koad127-B126] Tong J , Ren Z, Sun L, Zhou S, Yuan W, Hui Y, Ci D, Wang W, Fan L-M, Wu Z, et al ALBA proteins confer thermotolerance through stabilizing HSF messenger RNAs in cytoplasmic granules. Nat Plants. 2022:8(7):778–791. 10.1038/s41477-022-01175-1 35817823

[koad127-B127] Vequi-Suplicy CC , Riske KA, Knorr RL, Dimova R. Vesicles with charged domains. Biochim Biophys Acta. 2010:1798(7):1338–1347. 10.1016/j.bbamem.2009.12.023 20044978

[koad127-B128] Vernon RM , Chong PA, Tsang B, Kim TH, Bah A, Farber P, Lin H, Forman-Kay JD. Pi-Pi contacts are an overlooked protein feature relevant to phase separation. Elife. 2018:7:e31486. 10.7554/eLife.31486 PMC5847340 29424691

[koad127-B129] Wang J , Choi J-M, Holehouse AS, Lee HO, Zhang X, Jahnel M, Maharana S, Lemaitre R, Pozniakovsky A, Drechsel D, et al A molecular grammar governing the driving forces for phase separation of prion-like RNA binding proteins. Cell. 2018:174(3):688–699.e16. 10.1016/j.cell.2018.06.006 29961577 PMC6063760

[koad127-B130] Wang L , Tsuda K, Truman W, Sato M, Nguyen LV, Katagiri F, Glazebrook J. CBP60g and SARD1 play partially redundant critical roles in salicylic acid signaling. Plant J. 2011:67(6):1029–1041. 10.1111/j.1365-313X.2011.04655.x 21615571

[koad127-B131] Wheeler JR , Matheny T, Jain S, Abrisch R, Parker R. Distinct stages in stress granule assembly and disassembly. Elife. 2016:5. 10.7554/eLife.18413 PMC5014549 27602576

[koad127-B132] Wunder T , Mueller-Cajar O. Biomolecular condensates in photosynthesis and metabolism. Curr Opin Plant Biol. 2020:58:1–7. 10.1016/j.pbi.2020.08.006 32966943

[koad127-B133] Xu F , Wang L, Li Y, Shi J, Staiger D, Chen W, Wang L, Yu F. The Receptor Kinase FER Mediates Phase Separation of Glycine-Rich RNA-Binding Protein 7 to Confer Temperature Resilience in Arabidopsis. bioRxiv. 2022.

[koad127-B134] Yamasaki A , Alam JM, Noshiro D, Hirata E, Fujioka Y, Suzuki K, Ohsumi Y, Noda NN. Liquidity is a critical determinant for selective autophagy of protein condensates. Mol Cell. 2020:77(6):1163–1175.e9. 10.1016/j.molcel.2019.12.026 31995729

[koad127-B135] Yang P , Mathieu C, Kolaitis R-M, Zhang P, Messing J, Yurtsever U, Yang Z, Wu J, Li Y, Pan Q, et al G3BP1 is a tunable switch that triggers phase separation to assemble stress granules. Cell. 2020:181(2):325–345.e28. 10.1016/j.cell.2020.03.046 32302571 PMC7448383

[koad127-B136] Youn J-Y , Dyakov BJA, Zhang J, Knight JDR, Vernon RM, Forman-Kay JD, Gingras A-C. Properties of stress granule and P-body proteomes. Mol Cell. 2019:76(2):286–294. 10.1016/j.molcel.2019.09.014 31626750

[koad127-B137] Yu D , Song W, Tan EYJ, Liu L, Cao Y, Jirschitzka J, Li E, Logemann E, Xu C, Huang S, et al TIR domains of plant immune receptors are 2′,3′-cAMP/cGMP synthetases mediating cell death. Cell. 2022:185(13):2370–2386.e18. 10.1016/j.cell.2022.04.032 35597242

[koad127-B138] Yuan F , Alimohamadi H, Bakka B, Trementozzi AN, Day KJ, Fawzi NL, Rangamani P, Stachowiak JC. Membrane bending by protein phase separation. Proc Natl Acad Sci U S A. 2021:118(11):e2017435118. 10.1073/pnas.2017435118 PMC7980387 33688043

[koad127-B139] Zavaliev R , Mohan R, Chen T, Dong X. Formation of NPR1 condensates promotes cell survival during the plant immune response. Cell. 2020:182(5):1093–1108.e18. 10.1016/j.cell.2020.07.016 32810437 PMC7484032

[koad127-B140] Zhang Y , Sampathkumar A, Kerber SM, Swart C, Hille C, Seerangan K, Graf A, Sweetlove L, Fernie AR. A moonlighting role for enzymes of glycolysis in the co-localization of mitochondria and chloroplasts. Nat Commun. 2020:11(1):4509. 10.1038/s41467-020-18234-w 32908151 PMC7481185

[koad127-B141] Zhu S , Gu J, Yao J, Li Y, Zhang Z, Xia W, Wang Z, Gui X, Li L, Li D, et al Liquid-liquid phase separation of RBGD2/4 is required for heat stress resistance in *Arabidopsis*. Dev Cell. 2022:57(5):583–597.e6. 10.1016/j.devcel.2022.02.005 35231447

